# Programming of intestinal homeostasis in male rat offspring after maternal exposure to chlorpyrifos and/or to a high fat diet

**DOI:** 10.1038/s41598-021-90981-2

**Published:** 2021-06-01

**Authors:** Marion Guibourdenche, Hiba El Khayat El Sabbouri, Narimane Djekkoun, Hafida Khorsi-Cauet, Véronique Bach, Pauline M. Anton, Jérôme Gay-Quéheillard

**Affiliations:** 1 PériTox, Périnatalité & Risques Toxiques, UMR-I 01, UPJV/INERIS, Université Picardie Jules Verne, CURS, Présidence UPJV, Chemin du Thil, 80025 Amiens, France; 2 Institut Polytechnique UniLaSalle, Université d’Artois, ULR 7519, 19 rue Pierre Waguet, BP 30313, 60026 Beauvais, France

**Keywords:** Gastroenterology, Homeostasis

## Abstract

Alteration of programming of the intestinal wall maturation may be responsible for non-communicable chronic diseases in adulthood. It may originate from prenatal exposure of mothers to deleterious environmental factors such as pesticides or western diet. This work was undertaken to determine whether disturbances of the digestive tract function and of innate immunity of offspring at adulthood could be due to maternal exposure to a pesticide, chlorpyrifos (CPF) and a High Fat Diet (HFD) starting 4 months before gestation and lasting until weaning of offspring. Fifty-one male Wistar rats coming from 4 groups of dams exposed to CPF, HFD, both and control were followed from birth to 8 weeks of age. They were fed standard chow and received no treatment. The maternal pesticide exposure slows down fetal and postnatal weight gain without histological injuries of the gut mucosa. CPF or HFD both induced modifications of tight junctions and mucins genes expressions without inducing an increase in epithelial permeability or an inflammatory state. Co-exposure to both CPF and HFD did not exacerbate the effects observed with each factor separately. Despite the lack of direct contact except through breast milk until weaning, CPF or HFD maternal exposure have demonstrated preliminary gut barrier impacts on offspring.

## Introduction

Settlement and maturation of a healthy gut is based on a complex series of events starting in utero and continuing beyond birth, suckling and weaning periods^1^. It is based on genetic programming and modulated by epigenetic events^2^ which take place mainly during the first 1000 days of life. Then, due to its immaturity and thus to a higher intestinal permeability, the mucosal barrier of the gastrointestinal system of neonates and young infants can be more easily penetrated by macromolecules than that of older individuals^3^ and, as a consequence, may be more prone to injury and inflammation. This may encourage the transfer of antigens and other xenobiotics through the gut wall and may favor the onset of intestinal inflammation or gastrointestinal diseases^4^. As thus, altered intestinal barrier function during the perinatal period may have long-term health impacts at intestinal and/or systemic levels. Furthermore, it is now clearly established that environmental factors, among which diet or food contaminants to which mother and offspring may be exposed to, will influence the settlement and maturation of the intestinal tract of offspring through epigenetic events known to alter permanently their gut function and increasing their risk to develop non-communicable chronic diseases in adulthood. Pesticides, defined as substances used to control organisms considered as harmful for plants and animals, represent a very important category of food contaminants. The widest category of pesticides used worldwide are Organophosphates (OPs) although their use in France is currently decreasing. Our exposure to these pesticides is mainly oral, through the ingestion of pesticides residues in food (fruits and vegetables). This type of pesticides is known to induce an irreversible inhibition of acetylcholinesterase (AChE) activity, involved in acetylcholine recycling. As OPs and their metabolites easily cross the placenta, mothers might also expose their fetus all along gestation^5^ and the long-term effects of fetal or neonatal OPs exposure of offspring will have, through different mechanisms, an incidence on health later in life^6^. Among OPs, Chlorpyrifos-ethyl (CPF) exposure is known to affect the fetal nervous system^7,8^. It has been suggested that this molecule disrupts the embryonic development through perturbation of non-neuromuscular cell-to-cell interactions mediated by electrical events, such as intracellular ion concentration changes, similar to those involved in gametes interaction^9^. We have previously demonstrated a morphological modification of the intestinal villi, an alteration of the intestinal mucous barrier and stimulation of the intestine in pups exposed to pre- and post-natal CPF^10–12^. Other studies with in vitro approaches showed that CPF increases intestinal permeability^13,14^ but at this time little is known about the mechanisms by which CPF impacts the intestinal epithelial barrier, host's first line of defense against potential deleterious exogenous agents.

Furthermore, apart from pesticides, our diet may also represent a risk for our gut health. It is now well recognized that maternal diet and body composition before pregnancy has a great influence on her child future health^15–18^. Western diet may modify the digestive function and absorption of nutrients by influencing the architecture of the gut wall, mucus secretion or innate immunity^19^. However, the way by which mother’s diet may have an incidence on offspring gut settlement and maturation is, today, not understood. In this study, our aim was to determine whether a peri-gestational (pre-gestation, gestation and lactation) exposure of female rats to CPF and/or High Fat Diet (HFD) leads to disturbances of digestive function (paracellular permeability, mucus composition, intestinal morphology, inflammation, innate immunity…) in their male offspring at young adult age to qualify the importance of mother exposure on offspring programming of the gut maturation.

## Results

### Reduction of in utero growth but no digestive morphology modifications in male offspring from mothers exposed to CPF and/or HFD

**Figure 1:**
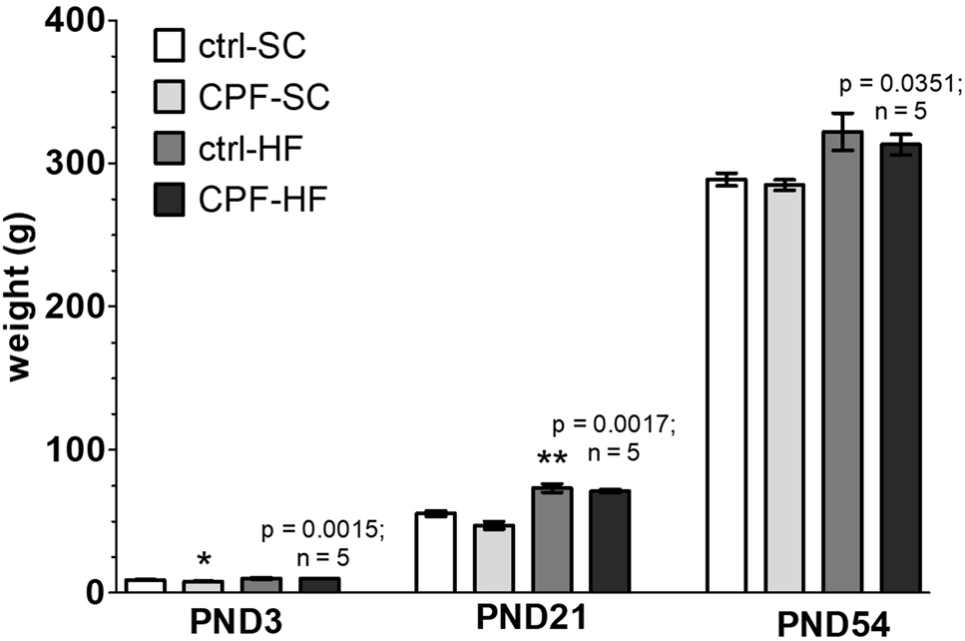
Body weight (g) at birth (PND3) after weaning (PND21) and at young adult age (PND54) of male offspring from CPF and/or HFD exposed mothers. Values are expressed as mean ± SEM (n = 5–20). *^,^**Significantly different (p < 0.05; p < 0.01) compared with ctrl-SC using the analysis of variance (ANOVA) and a subsequent post hoc Bonferroni test. *ctrl* control, *CPF* Chlorpyrifos, *HF* high fat, *SC* standard chow, *PND* postnatal day.

Male pups from mothers exposed to CPF had a significantly (p = 0.0196) lower body weight immediately after birth (Post-Natal Day 3—PND3; 7.99 g ± 0.11 g in the CPF-SC vs 9.04 g ± 0.32 g in the ctrl-SC; − 12%) (Fig. 1). By contrast, maternal HFD exposure significantly (p = 0.0317) increased body weight at weaning (PND21; 73.3 g ± 3.03 g in the ctrl-HF vs 55.5 g ± 1.90 g in the ctrl-SC; + 32%). It remained noticeably larger while not significant (p = 0.1932) until the end of the study (PND54; 322.2 g ± 12.99 g in the ctrl-HF vs 288.8 g ± 4.36 g in the ctrl-SC; + 12%). Furthermore, HFD exposure significantly (p = 0.0015; p = 0.0017 and p = 0.0351 respectively) rose CPF rats body weight not only immediately after birth (PND3; 10.09 g ± 0.13 g in the CPF-HF vs 7.99 g ± 0.11 g in the CPF-SC; + 26%) but also at weaning (PND21; 71.27 g ± 0.99 g in the CPF-HF vs 47.20 g ± 2.60 g in the CPF-SC; + 51%) and at early adulthood (PND54; 313.2 g ± 7.08 g in the CPF-HF vs 285.0 g ± 3.64 g in the CPF-SC; + 10%) (Fig. 1).

**Figure 2:**
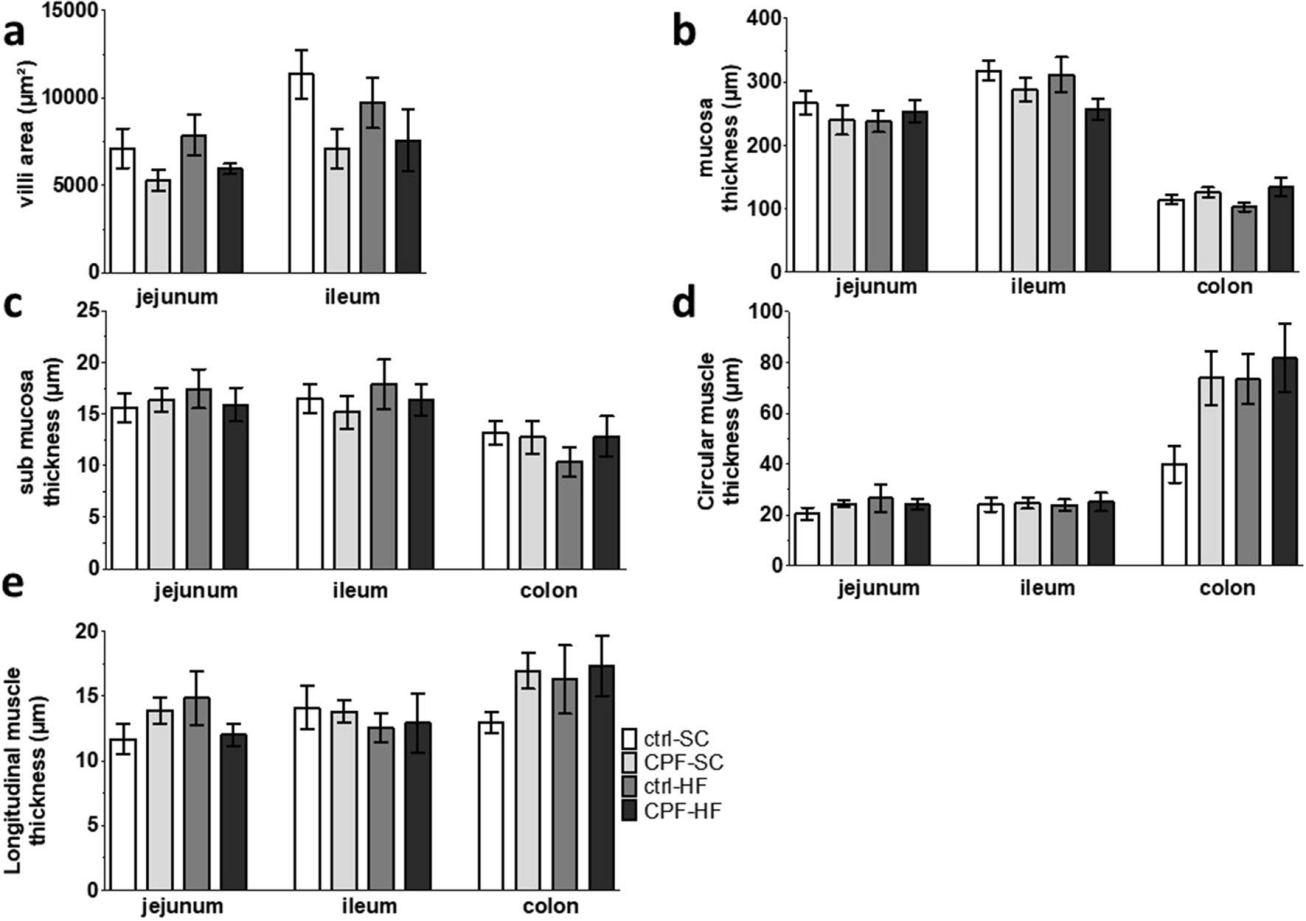
Histological measurements of digestive segments on male offspring at young adult age from CPF and/or HFD exposed mothers. (**a**) Villi area (µm^2^), (**b**) Mucosa thickness (µm), (**c**) Submucosa thickness (µm), (**d**) Circular muscle thickness (µm), (**e**) Longitudinal muscle thickness (µm) in jejunal, ileal and proximal colon segments. Values are expressed as the mean ± SEM (n = 5) using the analysis of variance (ANOVA) and a subsequent post hoc Bonferroni test. *ctrl* control, *CPF* Chlorpyrifos, *HF* high fat, *SC* standard chow.

Globally, in both the jejunum and the ileum at young adult age, area of the villi of the progeny from CPF exposed mothers were not significantly different (p > 0.05) from non-exposed mothers. Maternal HFD exposure induced a non-significant (p > 0.05) expansion for both the two segments compared with offspring from non-exposed mothers (Fig. 2a). However, we did not notice any significant difference (p > 0.05) between the offspring groups coming from mothers exposed to either CPF, HFD or both and the control groups regarding the digestive morphometric parameters studied: villi area, mucosa and submucosa thicknesses, circular and longitudinal muscle thicknesses (Fig. 2b–e).

### Partial alteration of markers of intestinal permeability in male offspring from mothers exposed to CPF and/or HFD

**Figure 3:**
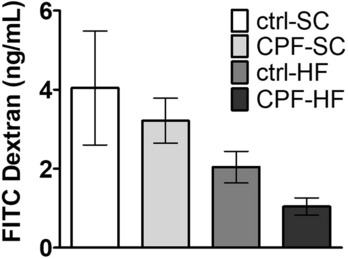
FITC dextran assays in plasma 2.5 h after oral administration in male offspring at young adult age from CPF and/or HFD exposed mothers. Values are expressed as the mean ± SEM (n = 5–20) using the analysis of variance (ANOVA) and a subsequent post hoc Bonferroni test. *ctrl* control, *CPF* Chlorpyrifos, *HF* high fat, *SC* standard chow, *FITC* fluorescein isothiocyanate.

FITC-dextran concentration measurement in plasma 2.5 h after oral administration was not associated with any significant difference (p > 0.05) between the different groups (ctrl-SC: 4.04 µg/mL; CPF-SC: 3.21 µg/mL; ctrl-HF: 2.04 µg/mL: CPF-HF: 1.04 µg/mL) (Fig. 3).

**Figure 4:**
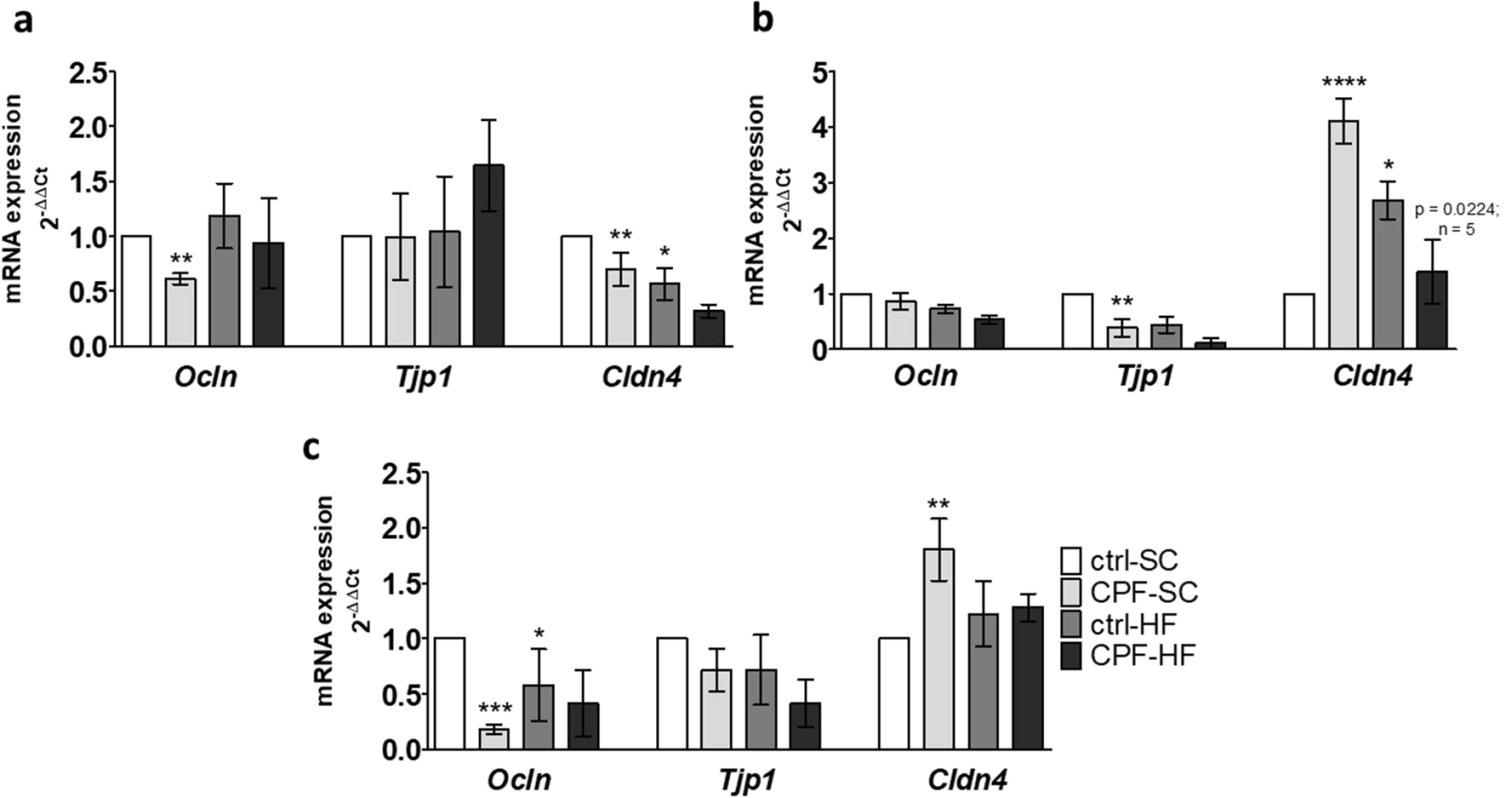
mRNA expression of genes coding for tight junctions’ proteins by RT-PCR in male offspring at young adult age from CPF and/or HFD exposed mothers. The mRNA levels of gene expression in (**a**) jejunum, (**b**) ileum, (**c**) proximal colon are expressed as fold induction over the control group set at 1. Values are expressed as mean ± SEM (n = 5–20) using the 2^−ΔΔCt^ calculation method with the housekeeping gene *Gapdh* for jejunum and ileum, and *Hprt1* for proximal colon segments. *^,^**^,^***^,^****Significantly different (p < 0.05; p < 0.01; p < 0.001; p < 0.0001) from ctrl-SC using the analysis of variance (ANOVA) and a subsequent post hoc Bonferroni test. *ctrl* control, *CPF* Chlorpyrifos, *HF* high fat, *SC* standard chow, *Cldn4* Claudin 4, *Ocln* occludin, *Tjp1/Zo-1 *Zonula occludens-1.

Jejunal mRNAs expressions of the *Ocln* and *Cldn4* genes were significantly (p = 0.0034 and p = 0.0038 respectively) decreased (0.39-fold and 0.30-fold respectively) in the CPF-SC, but it did not change the expression of *Tjp1* gene in this group (Fig. 4a). In the ileum, we noted a significantly reduced (p = 0.0010; 0.6-fold) mRNA expression for the *Tjp1* gene and a clear overexpression of *Cldn4* mRNA (p < 0.0001; 4.1-fold) in the CPF-SC by comparison to ctrl-SC (Fig. 4b). Then in the proximal colon, there was a significant decrease in the expression of the *Ocln* mRNA (p = 0.0007; 0.8-fold), and a significant overexpression of *Cldn4* mRNA in the CPF-SC compared to the ctrl-SC (p = 0.0064; 0.8-fold) (Fig. 4c).

Offspring from mothers exposed to HFD resulted in a significant decrease of jejunal expression of the *Cldn4* mRNA (p = 0.0178; 0.4-fold) as compared to the ctrl-SC (Fig. 4a). At last, in the proximal colon, maternal HFD exposure clearly reduced the expression of the *Ocln* gene (p = 0.0469; 0.4-fold) in the ctrl-HF by comparison to the ctrl-SC (Fig. 4c).

By contrast, we observed in the ileum from the CPF-HF, a significant reduction of the expression of *Cldn4* mRNA (p = 0.0224; 0.7-fold) by comparison to the CPF-SC (Fig. 4b).

### Modification of innate immunity reactivity and inflammatory markers in male offspring from mothers exposed to CPF and/or HFD

#### Absence of induction of an inflammatory reaction in the digestive tract on male offspring from mothers exposed to CPF and/or HFD

**Figure 5:**
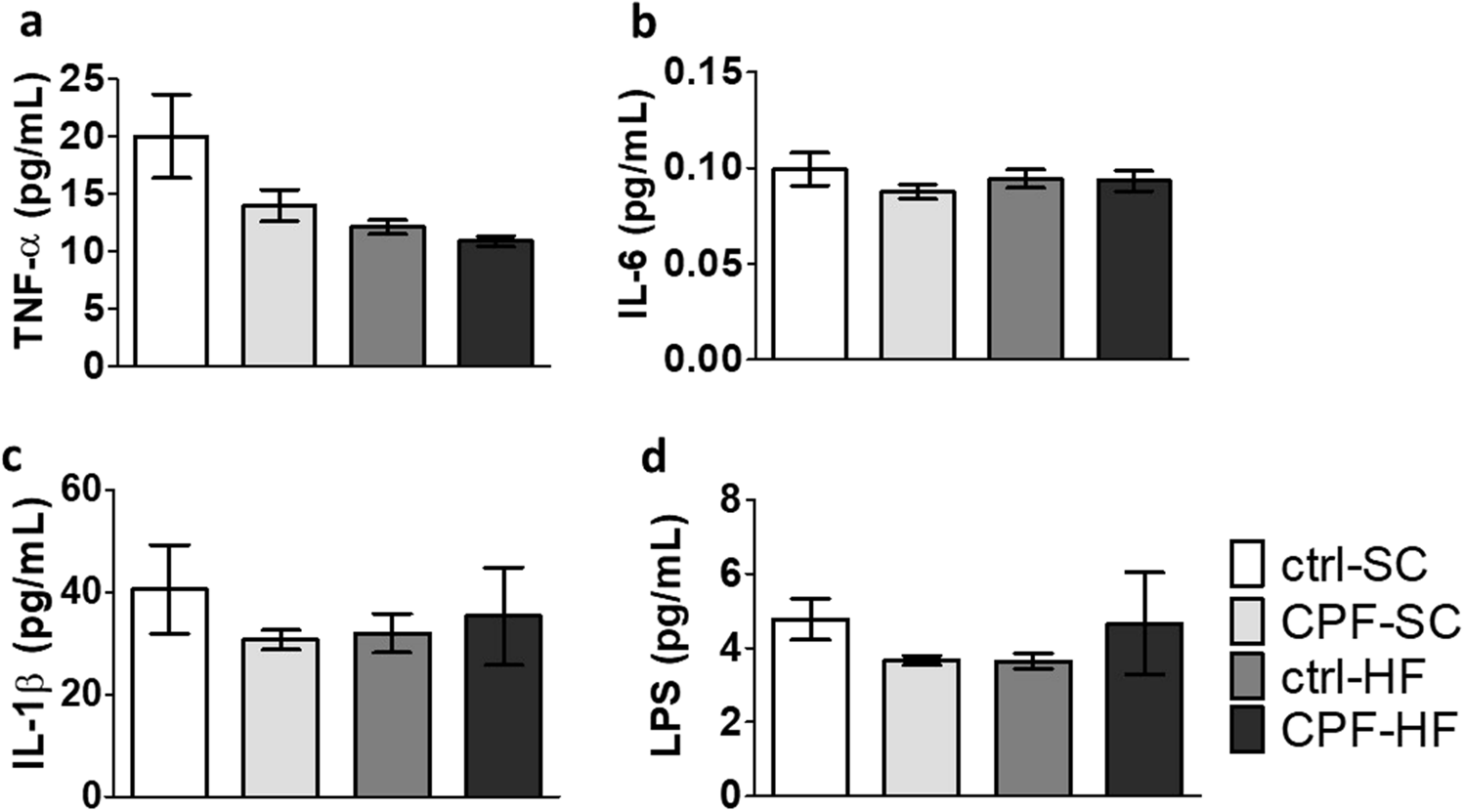
Blood pro-inflammatory cytokines and LPS levels (pg/mL) in male offspring at young adult age from CPF and/or HFD exposed mothers IL-6. Plasma levels of (**a**) TNF-α (**b**) IL-6 (**c**) IL-1β (**d**) LPS were measured. Values are expressed as the mean ± SEM (n = 5–20) using the analysis of variance (ANOVA) and a subsequent post hoc Bonferroni test. *ctrl* control, *CPF* Chlorpyrifos, *HF* high fat, *SC* standard chow, *IL-1b* Interleukin 1 beta, *IL-6* Interleukin 6, *LPS* lipopolysaccharides, *TNF-α* tumor necrosis factor alpha.

Whichever treatment received by mothers, plasma cytokines levels (IL-1β, IL-6 and TNF-α) measured at young adult age were not different (p > 0.05) (Fig. 5a–c). Additionally, as a marker of potential excessive bacterial products transfer through the intestinal epithelial barrier, and thus of permeability alteration, the plasma LPS assay in the 4 groups of offspring was not associated to significant differences (p > 0.05) at young adult age (Fig. 5d).

#### Alteration of innate immunity markers at the intestinal and colonic mucosal levels in male offspring from mothers exposed to CPF and/or HFD

**Figure 6:**
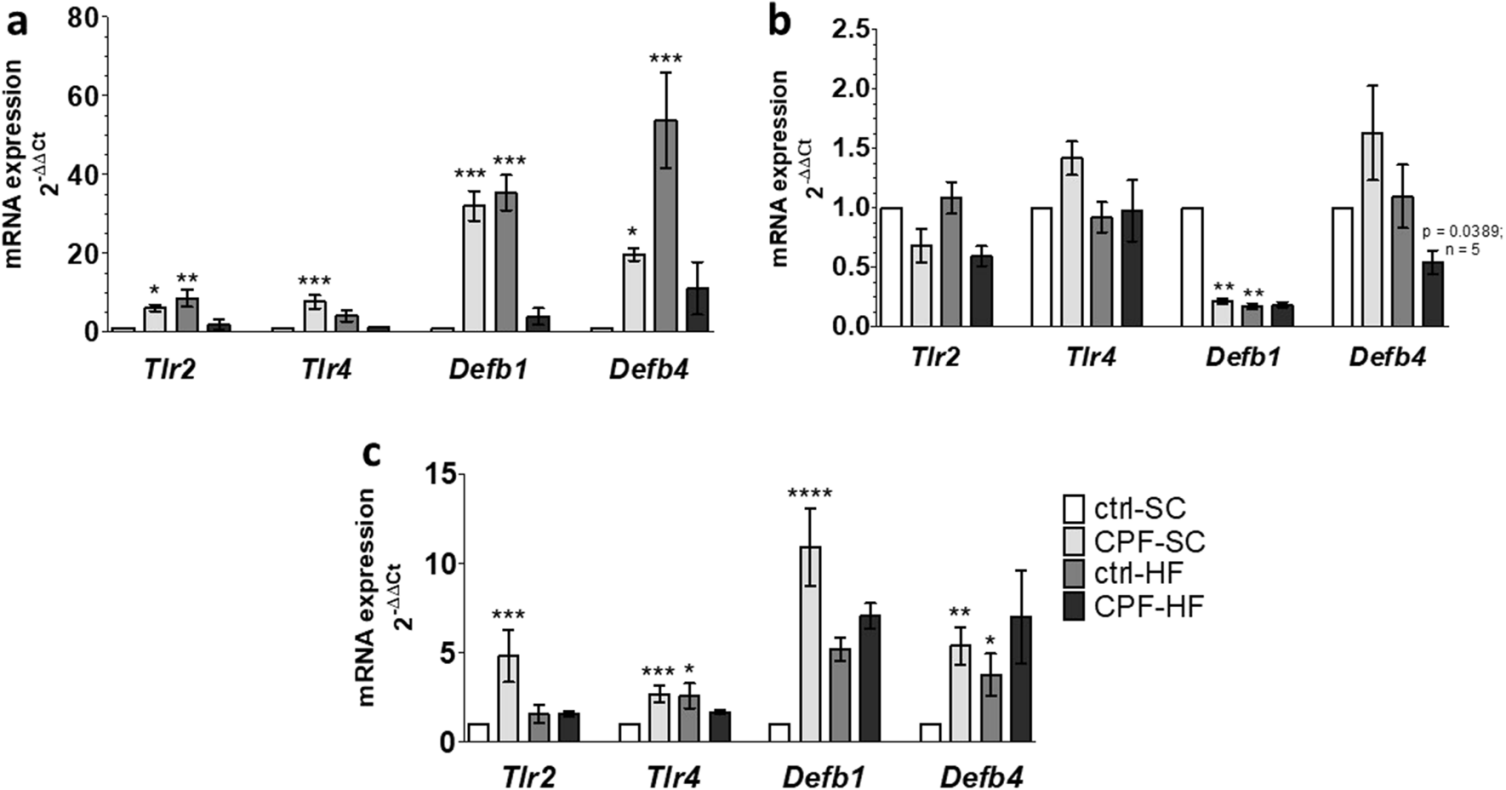
mRNA expression of genes coding for *Tlrs* and *Β Defensins* by RT-PCR in male offspring at young adult age from CPF and/or HFD exposed mothers. The mRNA levels of gene expression in (**a**) jejunum, (**b**) ileum, (**c**) proximal colon are expressed as fold induction over the control group set at 1. Values are expressed as mean ± SEM (n = 5–20) using the 2^−ΔΔCt^ calculation method with the housekeeping gene *Gapdh* for jejunum and ileum, and *Hprt1* for proximal colon segments^.^ *^,^**^,^***^,^****Significantly different (p < 0.05, p < 0.01; p < 0.001; p < 0.0001) from ctrl-SC using the analysis of variance (ANOVA) and a subsequent post hoc Bonferroni test. *ctrl* control, *CPF* Chlorpyrifos, *HF* high fat, *SC* standard chow, *Defb1* Defensin β1, *Defb4* Defensin β4, *Tlr2* Toll Like Receptor 2, *Tlr4* Toll Like Receptor 4.

At the jejunal level, maternal CPF exposure significantly (p = 0.0106 and p = 0.0001 respectively) increased (6.1-fold and 7.7-fold; respectively) the expression of not only *Tlr2* and *Tlr4* mRNA but also significantly (p = 0.0001 and p < 0.05 respectively) increased (32.0-fold and 18.9-fold respectively) the expression of *Defb1* and *Defb4* mRNA as compared to the ctrl-SC (Fig. 6a). By contrast, maternal CPF exposure was responsible for a significant decrease (p = 0.0025; 0.8-fold) of ileal expression of *Defb1* mRNA by comparison to the ctrl-SC (Fig. 6b). At last, maternal CPF exposure resulted in a significant (p = 0.0009; p = 0.0001, p < 0.0001 and p = 0.0012 respectively) increase of the proximal colic mRNA expression of *Tlr2*, *Tlr4*, *Defb1* and *Defb4* (3.90, 2.67, 10.9 and 5.4-fold respectively) genes by comparison to the ctrl-SC (Fig. 6c).

On the other hand, maternal HFD exposure was at the origin of a significant (p = 0.0039, p = 0.0009 and p < 0.001; respectively) increase of *Tlr2*, *Defb1* and *Defb4* (8.6, 35.3 and 53.8-fold, respectively) mRNA expression in the jejunum by comparison to the ctrl-SC (Fig. 6a). By contrast, in the ileum, a significant reduction (p = 0.0016; 0.8-fold) of mRNA expression of *Defb1* was observed compared to the ctrl-SC. In the proximal colon, maternal HFD exposure was associated to a significant (p = 0.0216 and p = 0.0467 respectively) increase of mRNA expression of *Tlr4* and *Defb4* (2.6 and 3.8-fold) as compared to ctrl-SC (Fig. 6c).

Co-exposure of mothers to CPF and HFD (CPF-HF) resulted in a significant downregulation (p = 0.0389; 0.7-fold) of *Defb4* mRNA expression in the ileum as compared to the CPF-SC (Fig. 6b).

#### Maternal CPF or HFD exposure downregulated mucins mRNA expressions in the progeny at young adult age

**Figure 7:**
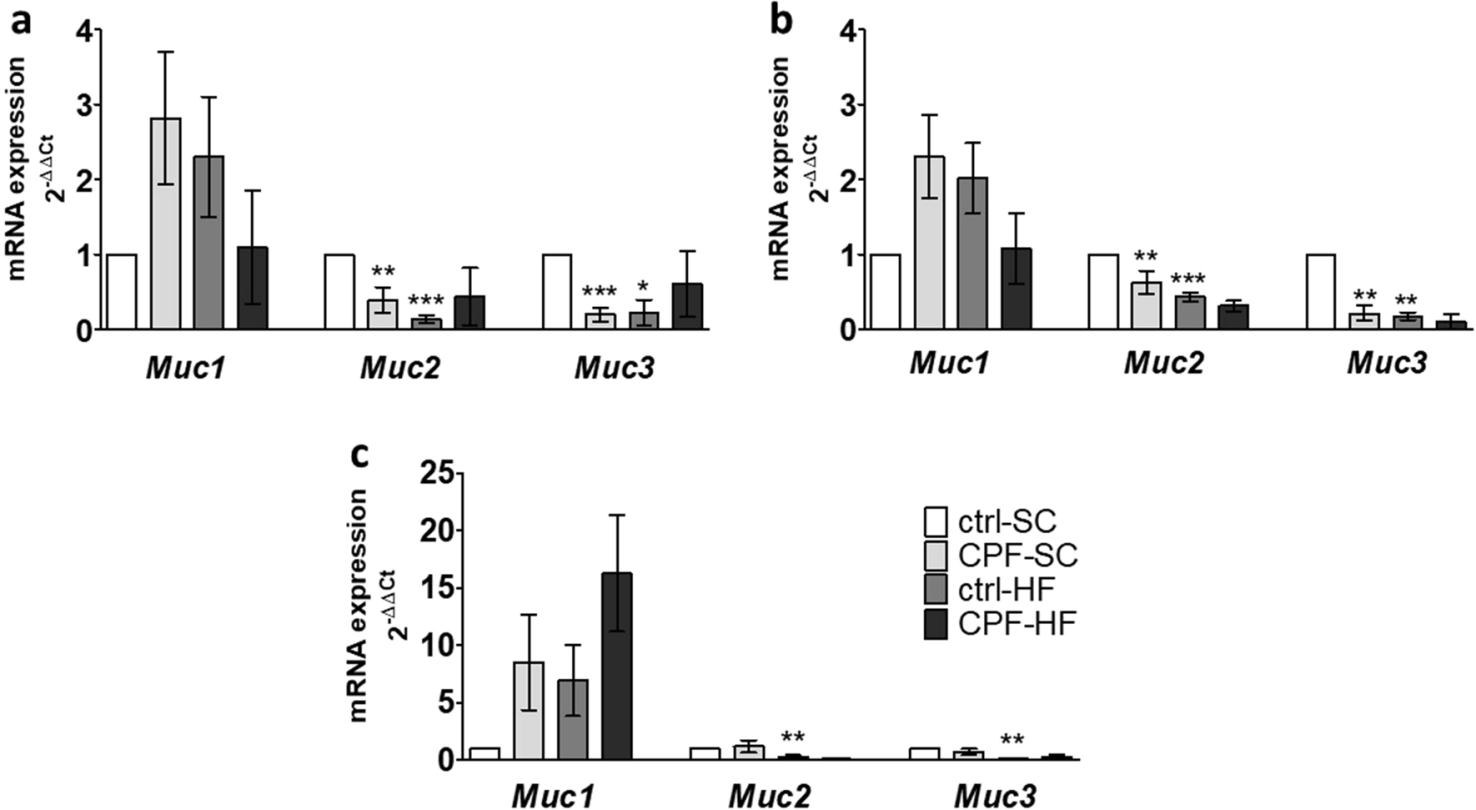
mRNA expression of genes coding for mucins’ proteins by RT-PCR in male offspring at young adult age from CPF and/or HFD exposed mothers. The mRNA levels of genes expression in (**a**) jejunum, (**b**) ileum, (**c**) proximal colon are expressed as fold induction over the control group set at 1. Values are expressed as mean ± SEM (n = 5–20) using the 2^−ΔΔCt^ calculation method with the housekeeping gene *Gapdh* for jejunum and ileum, and *Hprt1* for proximal colon segments. *^,^**^,^***Significantly different (p < 0.05; p < 0.01; p < 0.001) from ctrl-SC using the analysis of variance (ANOVA) and a subsequent post hoc Bonferroni test. *ctrl* control, *CPF* Chlorpyrifos, *HF* high fat, *SC* standard chow, *Muc1* Mucin 1, *Muc2* Mucin 2, *Muc3* Mucin 3.

Maternal exposure to CPF resulted in a significant downregulation of the expression of *Muc2* and *Muc3* genes both in the jejunum (0.6-fold; p = 0.0058 and p < 0.0001; 0.8-fold respectively) and the ileum (0.4-fold; p = 0.0016 and 0.8-fold; p = 0.0039 respectively) in the CPF-SC as compared to the ctrl-SC (Fig. 7a,b). By contrast, we did not notice, in this group of animals, any difference in the expression of genes coding for mucins in the proximal colon (Fig. 7c).

On the other hand, mothers exposure to HFD was associated to a significant lower expression of both *Muc2* and *Muc3* mRNA in the jejunum (0.8-fold, p < 0.0001 and 0.8-fold, p = 0.0163, respectively), in the ileum (0.6-fold, p = 0.0009 and 0.8-fold, p = 0.0062, respectively) and in the proximal colon (0.7-fold, p = 0.0085 and 0.9-fold, p = 0.0041 respectively) in the ctrl-HF as compared to the ctrl-SC (Fig. 7a–c).

#### Specific upregulation of goblet cell number in the ileum of offspring from mothers exposed to CPF

**Figure 8:**
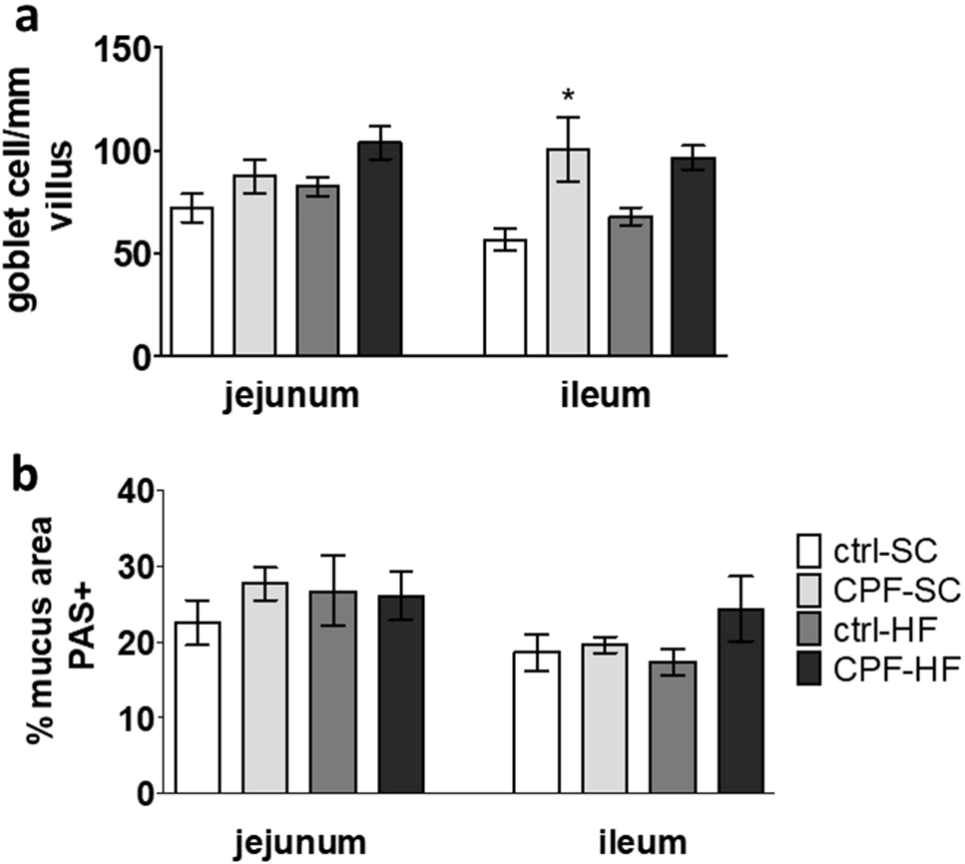
Histological measurements of digestive segments in male offspring at young adult age from CPF and/or HFD exposed mothers. (**a**) Goblet cells count/mm villus and (**b**) % of mucus area PAS^+^ in jejunal and ileal segments. Values are expressed as the mean ± SEM (n = 5–20) *Significantly different (p < 0.05) from ctrl-SC using the analysis of variance (ANOVA) and a subsequent post hoc Bonferroni test. *ctrl* control, *CPF* Chlorpyrifos, *HF* high fat, *SC* standard chow, *PAS* periodic acid schiff.

In the ileum of CPF-SC, we noticed a significant increase (+ 78%; p = 0.0215) of the number of goblet cells by mm of villus (100.6 ± 15.68 *vs* 56.5 ± 5.40 goblet cells/mm in the ctrl-SC) (Fig. 8a). By contrast, no significant difference was highlighted in the jejunum (Fig. 8a). Furthermore, evaluation of the secretion of mucus (PAS staining) did not point at any significant difference between the groups (Fig. 8b).

## Discussion

In this study, we sought to determine the consequences of maternal long-term exposure (pre-gestation, gestation and lactation) to deleterious environmental factors such as pesticide residues associated to an unbalanced diet on the programming of body growth and of the intestinal wall function of its male offspring. A repeated contact with pesticides or through maternal unbalanced diet may have strong health consequences. Indeed, measurable pups lactational exposure to CPF (oral, 1 mg/kg/day) was previously confirmed, CPF being concentrated in the milk^20^. In addition, rat relevant offspring NOAEL for CPF is 1 mg/kg/day which is the dose used in the study^21^, To guarantee a homogenous exposure of animals to CPF, we chose to expose animals to the pesticide by orogastric gavage to mimic the oral route which is the most frequent route of exposure to this pesticide. This technique won’t generate neither stress nor behavioural or metabolic consequences on the mother or its progeny when realized by an experienced person^22^. The nutritional composition of breast milk could also be affected by maternal obesity or diet during pregnancy and lactation. Changes in milk composition of lipids during lactation might contribute to the long-term metabolic changes induced by maternal HFD^23^.

We have shown that chronic maternal exposure to CPF reduces in utero growth. In link with these results, we studied potential disturbances of the digestive wall architecture of the offspring. We observed the consequences of these two factors at PND60 which corresponds to young adult rats^24^ and is right after becoming sexually mature^25^. Indeed, the development of the gastrointestinal tract starts in utero and maturation continues beyond weaning^1^. However, in our model, we did not notice any difference between groups from mothers exposed to CPF and/or HFD and the control group regarding the digestive morphometry. This is surprising since both CPF and HFD are known to induce inhibition of AChE^26^ and since we have recently confirmed that AChE activity was reduced in young adult male progeny (PND60) on this model^27^. Indeed, AChE inhibition may cause structural defects in the digestive tract, as thanks to its expression in gut endoderm of the embryo, it plays a role in intestinal morphogenesis^28^. As thus, these effects may impact nutrients absorption and induce growth deficiency. Furthermore, we have previously published that maternal CPF exposure (1 and 5 mg/kg/day from gestation until PND60) delayed maturation of the digestive system (villi height and width, epithelium thickness and muscular layers thickness). But the characteristics mentioned above were evident at PND21 at the highest dose used and when the offspring were directly exposed to the pesticide^11^. In addition, maternal obesity (HFD exposure starting 8 weeks before pregnancy and lasting until the end of lactation) was at the origin of a reduction in villi height and of an increase of crypt depth in ileum of female mice offspring^29^. The authors suggested that these impacts may come from a reduction from enhanced epithelial turnover due to observed inflammation. Such a difference may come from differences in the experimental model, as Xue et al. studied the effects of a maternal HFD exposure for 8 weeks on female mice at 16 weeks of age. In our model, species used was rats and the exposure timing was 16 weeks before mating, and the pups were studied at the age of 8 weeks.

Another stricking point is the low fertility of CPF-HF mothers that was not expected. Indeed, although HFD is described as impairing female reproductive function through an alteration of the hypothalamic–pituitary–ovarian axis functionality on rats^30^ and CPF as inducing reproductive problems (changed the sperm, serum hormones, oxidative stress in the testis, and enzyme activity related to spermatogenesis in rats)^31^, we did not observe such consequences in our model. Since animals were raised in the same environment, the only explanation may be linked to the cumulative effect of both the deleterious factors studied. To clarify this point, further studies will have to be performed to evaluate the cumulative consequences of CPF and HDF on the reproductive function with special attention to putative follicular atresia in the ovaries of females.

Indeed, intestinal barrier may be disturbed at the mucus layer level, and particularly through its mucin composition. As a matter of facts, secretory mucins form a protective mucus gel barrier on the epithelia against potentially deleterious environment present in the lumen of the gastro-intestinal tract^32^. In our model, maternal exposure to CPF or HFD decreased, in their offspring, the expression of *Muc2* and *Muc3* responsible for mucus properties and secretion, although we did not observe any modification of mucus release in all the intestinal segments studied. There still remains to be established whether it results in a modification of protein expression and functionality but also to see which mechanism will therefore be at the origin of these genes expressions modifications. This reduction in mucins expression is associated with an increase in the number of mucus cells per mm of villus only in the offspring’s ileum from CPF exposed mothers. These data are contradictory with a previous study of our laboratory in which we revealed that chronic maternal and postnatal CPF-exposure were associated with a higher expression of *Muc2* in the ileum and the proximal colon and that the effects were more relevant at weaning. Such a difference may be explained by the fact that progeny was directly exposed after weaning to CPF and to a higher dose (5 mg/kg/day)^11^.

HFD maternal exposure induced the same deleterious results as those observed with CPF. This is corroborated by another work showing that, in an in vitro model of epithelial/mucus cells co-culture, an excess of nutrients increased mucus production and intestinal permeability without inducing any inflammation^33^. The absence of effects on intestinal permeability in our model may be due to a more complex architecture of the epithelium in vivo. Furthermore, in another experiment, HFD-induced maternal obesity reduced the density of goblet cells per villus observed with Alcian blue staining in the ileum of 16 weeks old female offspring^29^. One of the explanation could be that the alteration of mucus barrier may not be significant at PND60 (reduction of secreted mucins but not of mucus thickness) but could become significant later in life (thinner mucus layer) rendering the animals more prone to develop gut inflammation.

In this study, we also managed to evaluate the consequences of maternal exposure to a pesticide residue (CPF) together with an imbalanced (HF) diet on the intestinal mucosal barrier function. We observed changes in the expression of genes coding for TJ proteins, which maintain the strength of the mucosal barrier, a strong component of the regulation of paracellular permeability in relation to maternal exposure. In offspring from mothers exposed to CPF, we evidenced a significantly reduced expression of *Ocln and Cldn4* in the jejunum, of *Tjp1* in the ileum and of *Ocln* in proximal colon, while *Cldn4* was overexpressed in both the ileum and the proximal colon. However, we did not evidence any impact neither on paracellular permeability after measuring plasma-dextran nor on the plasma LPS level, a marker of excessive bacterial products passage through the intestinal epithelial barrier in these offspring. This is not surprising since in normal physiological conditions, these genes coding for TJ proteins encounter variations in their respective expression that appears to be dependent on the intestinal segment. As we have already described, all junctional proteins are not similarly influenced by the experimental conditions and this is in accordance with the fine regulation of junctional complexes^12^. Furthermore, this study showed a rise in paracellular permeability at weaning (PND21), when the digestive system is still immature, but no more at PND60^12^. This event was associated with a disturbance in the expression and/or the localization of TJ and with bacterial translocation and dysbiosis at weaning and at young adulthood^11,12^.

Following HFD maternal exposure, a reduction of the expression of *Cldn4* in the jejunum and *Ocln* in the proximal colon, together with an ileal overexpression of *Cldn4* were observed in male offspring. It is not noteworthy that maternal HFD led to changes in the same direction as CPF effects without, however, exacerbating them. The observed impacts might only be predictive of more severe alterations later in life considering that 8 weeks old was a probably too early experimental timing to observe such effects. We could then suggest that may inhibit the bioactivation of CPF into its metabolites by decreasing cytochrome P450 (CYP450) isoforms^26,34^. Moreover, since at birth, the bioactivation of CPF, catalyzed by CYP, is much higher than the detoxification^35^, this may indicate a role for a downregulating effect of HFD on CPF toxic effects^36^.

Indeed, perturbations of gene expression of TJ proteins were not associated to any clear alteration of intestinal paracellular permeability. These results are in agreement with previous results demonstrating a decrease of *Zo-2* and *Zo-3* gene expression and an increase of *Cldn2* at both mRNA and protein levels and an increase of plasma LPS level while they did not observe any alteration of intestinal permeability (plasma FITC) on 16 weeks old female mice offspring after maternal HFD^29^. This difference on plasma LPS level may be explained by the fact that, in their work, authors studied female rodent. Indeed, females are known to have a greater magnitude of gut permeability than male^37^.

Knowing that the presence of pro-inflammatory cytokines in the intestinal compartment could be able to modulate the expression of claudins proteins orchestrated by transcriptional regulation, we then evaluated the effects of mother exposure to CPF and HFD on gut inflammatory markers in their offspring. We observed that *Tlr* and *Defensin* gene expressions were increased in the jejunum and the proximal colon of offspring whereas we did not observe any inflammatory signs as shown by the plasma cytokines levels in the different groups of animals whichever treatment considered. It is well established that TLRs recognize harmful molecules from pathogens and activate the innate immune system and thus the adaptive immune response (for review see Ref.^38^). For example, TLR2 and TLR4 specifically recognize bacterial LPS and their activation mainly lead to the synthesis of pro-inflammatory cytokines and chemokines^39^. Their expression levels are increased in macrophages and dendritic cells during inflammation^40^. Gut immune system is altered under HFD in link with induced inflammation. Furthermore, TLR4 upregulation may come from gut inflammation induced by HFD^41^. This upregulation is known to alter gut microbiota composition which is responsible for a disturbance of the homeostasis and of the development of the body's immune defense against some bacteria at the origin of the increased production of β defensins^42^. We were unable to detect any obvious sign of inflammation nor any morphological defect or innate immune cells infiltration at the intestinal level. One of the reason why we saw no upregulation of pro-inflammatory cytokines may be linked with the fact that we measured circulating cytokines but not tissular ones. We cannot exclude, however, that pro-inflammatory cytokines may be moderately released in the mucosa inducing a modification of TJs composition and influence their overall balance and thus the barrier function^43^.

**Figure 9:**
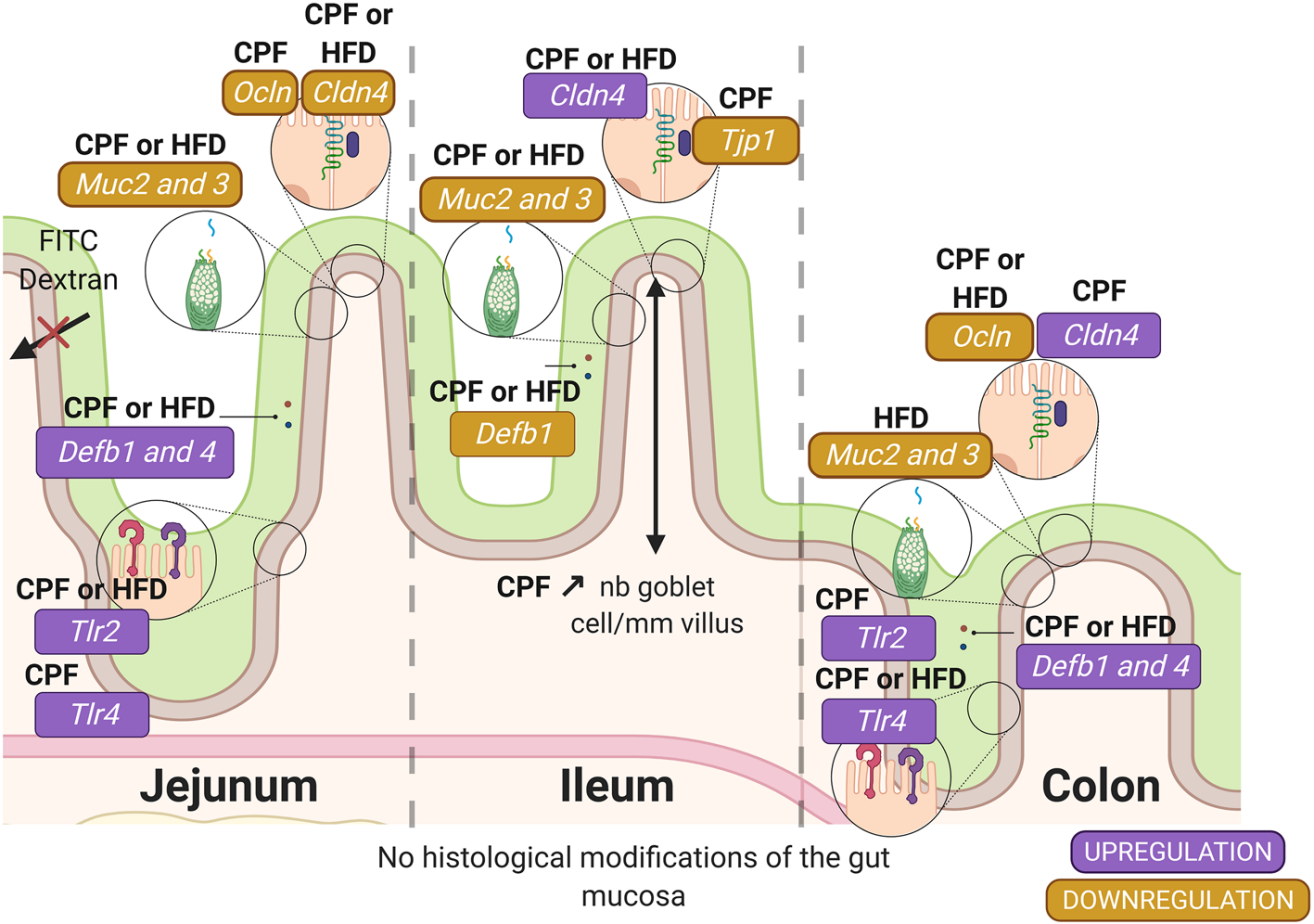
Effect on intestinal barrier in progeny from mothers submitted to CPF in the absence or presence of HFD. Created with BioRender.com; *CPF* Chlorpyrifos, *HFD* high fat diet, *Cldn4* Claudin 4, *Defb1* Defensin β1, *Defb4* Defensin β4, *Muc1* Mucin 1, *Muc2* Mucin 2, *Muc3* Mucin 3, *Ocln* Occludin, *Tjp1/Zo-1 *Zonula occludens-1, *Tlr2* Toll Like Receptor 2, *Tlr4* Toll Like Receptor 4.

This study was aimed at identifying the potential impacts on the intestinal barrier and integrity of the offspring of a harmful maternal environment, consisting in a food rich in saturated fat associated to pesticide residues during the perinatal period. Even in the absence of direct contact except through blood during the in utero period and breast milk until weaning, CPF or HFD maternal exposure started to modulate markers of gut barrier function in the progeny. Furthermore, maternal CPF exposure slowed down fetal and postnatal growth without inducing major histological modifications of the gut mucosa. CPF or HFD also partly altered some components of intestinal barrier function as observed by the decrease in expression of genes involved in maintaining tight junctions and mucus composition, despite of any observed perturbations in intestinal permeability known to be at the origin of inflammation (Fig. 9). A maternal HFD did not exacerbate CPF’s observed effects. We can also suggest that the observed overexpression of TLRs in this model is probably a sign of intestinal dysbiosis and that the increase of mRNA expression of defensins might probably be due to bacterial components specific to TLR increased expression on enterocytes. This will have to be confirmed by the analysis of gut microbiota composition of the progeny. Altogether, results play in favor of a role of pesticide residues and western diet in the alteration of the settlement of gut barrier function in the progeny. If confirmed at a later stage, this could contribute to the characterization of early stage markers to predict the consequences of perinatal exposure to environmental factors on the settlement of chronic non-communicable diseases later in life.

## Methods

### Animal housing

Fifty-one male Wistar rats coming from sixteen females (Janvier laboratory—Saint Berthevin, France) were used. Animals were pair housed in stainless steel and enriched cage (presence of roller, sizzle pad and chips) in a controlled air-temperature (23 ± 1 °C). The room had a constant relative humidity of 26% and artificial lighting (12-h night/light cycle)^27^.

Animals welfare was monitored daily. All experiments were performed according to the protocol reviewed by the local ethic committee (C2EA-96) and approved by the French Ministry of Research (reference APAFIS # 8207-2016121322563594 v2). All animals experiments comply with EU Directive 2010/63/EU and with the ARRIVE guidelines (Animal Research: Reporting of In Vivo Experiments).

### Animals experiment

**Figure 10:**
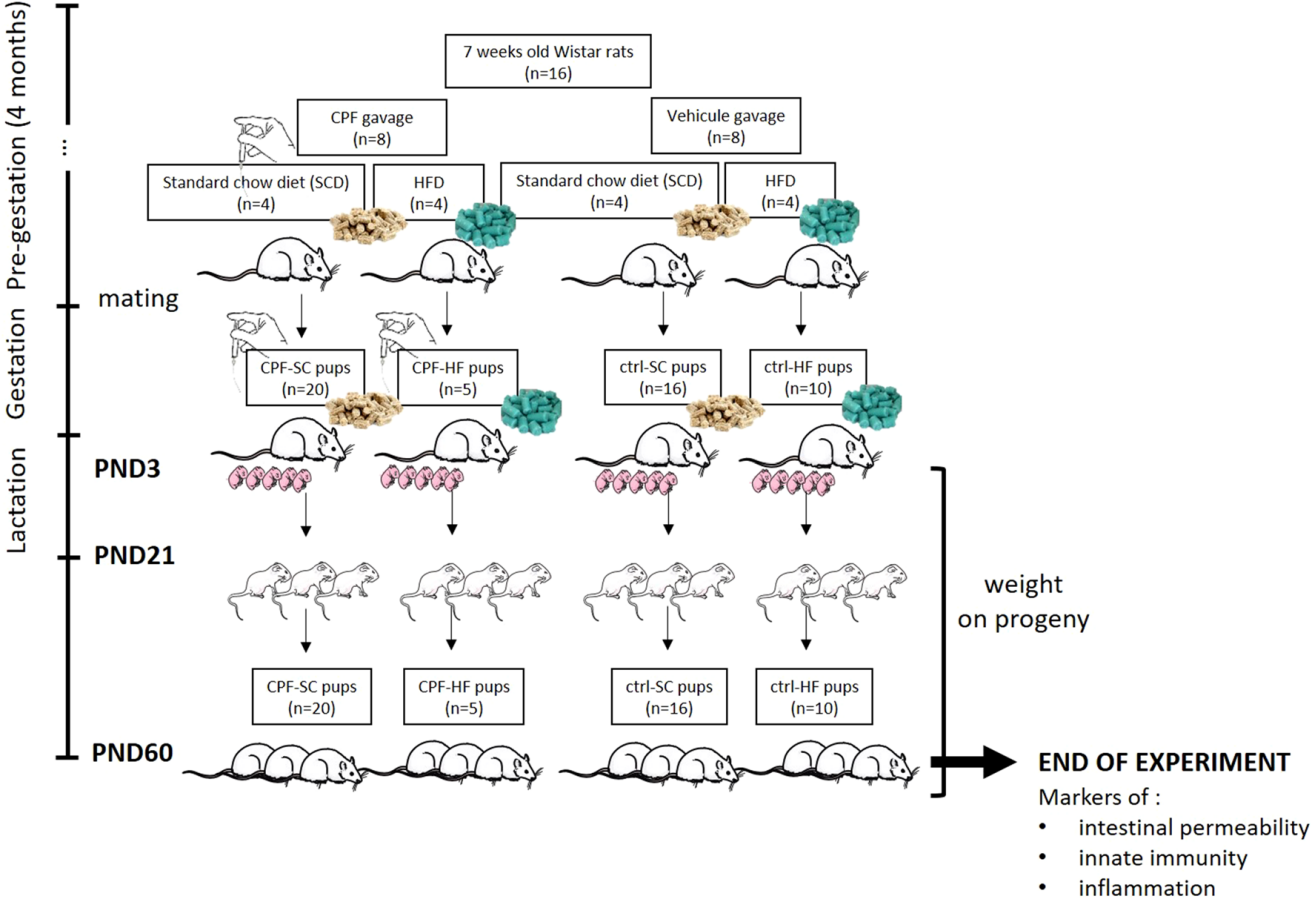
Experimental procedure. Created with BioRender.com*;* on the left-hand side of the figure is indicated the chronology of the experiments and on the main panel the events to which dams and progeny were submitted. *ctrl* control, *CPF* chlorpyrifos, *HFD* high fat diet, *SCD* standard chow diet, *PND* postnatal day.

Male offspring coming from mothers submitted to different environmental conditions were followed from birth until early adult age (PND60). They received no treatment and had access to standard chow and water ad libitum (Fig. 10) throughout the study. After one week of acclimation, the 16 mothers (225 ± 17 g—8 weeks-old), were assigned randomly to different experimental conditions. Females were divided into 2 groups of 8 respectively receiving either CPF (O. O-diethyl-O-(3.5.6-trichloro-2-pyridinyl) phosphorothioate solution (1 mg/kg/day po—1 mL/kg/day) (LGC Standards Laboratory, Molsheim, France) or its vehicle rapeseed oil (1 mL/kg/day) (MP Biomedicals, Illkirch, France) (Fig. 10). Each group was further divided in 2 subgroups receiving either a high fat (HF) diet (60% energy from fat) (D12492; laboratory Ssniff Spezialdiäten GmbH, Soest, Germany) (n = 4/group) or a standard chow (SC) diet (maintenance chow—Serlab 3436, or breeding chow during gestation and lactation—Serlab 3336) (Serlab, Montataire, France) (Fig. 10). This model has been shown to induce metabolic alterations (increased body weight (BW), hyperinsulinemia, programming of adiposity…) in the offspring at adulthood (7 months of age)^44^. With this model, standard rodent chow was used as control diet because a recent study showed that both a purified low-fat diet or a regular chow may be appropriate as controls for an HFD study^45^. In their work, authors demonstrated that the use of these diets during 16–18 weeks on mice had similar effects on phenotypic (weight gain), metabolic (glucose tolerance, levels of high-density lipoprotein, triglycerides, plasma cytokines and adipocytokines) and behavior outcomes. Furthermore, in our study, we did not study the incidence of these diets on mothers but rather specifically focused on their offspring which all were under Standard Chow Diet (SCD). Features of the diets are described in Supplementary Table S1.

After 4 months of exposure (24 weeks old), the 16 females (316 ± 33 g) were mated. Pregnancy was confirmed by the presence of spermatozoa and/or plug in the vagina checked twice with a smear on the following early morning. After fertilization, pregnant females were individually housed in clean plastic cages and treatments and diets were pursued [HFD vs SCD; CPF vs rapeseed oil (ctrl)] until the end of lactation. The day of parturition was considered as post-natal day 1 (PND1). A litter was composed of a mean of 10 ± 5 pups (4 ± 3 male pups per litter). The litter size was not significantly different between groups. Male pups represented 54% of the total offspring. Legs of each pup were tattooed with a veterinary ink (Centravet, Nancy, France) for individual identification. Pups were counted, sexed and weighed at PND3, to limit possible early-life maternal separation stressor; they were not weighed at PND1. Pups were weighed every 3 days until PND54. Animals were sacrificed at PND60, when young adults^24^. At weaning (PND21), pups were separated from their mothers and fed tap water and SC diet (SCD) (Serlab 3436), ad libitum (Fig. 10). In order to avoid potentially confounding factors such as menstrual cycles and the impact of hormones on physiological functions, only male pups were studied. They were all coming from one of the following groups:

- Offspring ctrl-SC group: maternal exposure to SCD without CPF (n = 16)
- Offspring CPF-SC group: maternal exposure to SCD with CPF (n = 20)
- Offspring ctrl-HF group: maternal exposure to HFD without CPF (n = 10)
- Offspring CPF-HF group: maternal exposure to both HFD and CPF (n = 5)

Because of some fertility problems, the number of male pups from the co-exposition group (CPF-HF) was lower than other groups.

The night before sacrifice, animals were fasted. Pups were euthanized with a single intraperitoneal of sodic pentobarbital (EXAGON 140 mg/kg—0.35 mL/kg) (Axience SAS, Pantin, France). Blood was drawn and plasma was obtained by centrifugation (3000*g*, 15 min), immediately aliquoted and stored at − 80 °C until further analysis to determine plasma Fluorescein isothiocyanate (FITC)-dextran, cytokines levels and Lipopolysaccharides (LPS) concentrations. Intestinal samples (jejunum, ileum and proximal colon) were collected under sterile conditions. For gene expression study, at harvest, the digestive tissue samples were deeped in a RNA later Stabilization Solution (Life Technologies, Thermo Fisher Scientific, Courtaboeuf, France), a non-toxic tissue storage reagent allowing rapid tissue permeation and cellular RNA stabilization and protection. After a short stabilization period, the samples were immediately frozen at − 80 °C until RNA extraction procedure. Another set of digestive tissue sample was taken in order to proceed to mucins production and histopathological analysis and deeped into a Carnoy’s fixative solution.

### Evaluation of intestinal permeability modifications in male offspring from mothers submitted to CPF and/or HFD

#### FITC Dextran transfer into blood circulation as a marker of paracellular permeability

Intestinal permeability was estimated from the amount of FITC-dextran that had crossed the intestinal epithelial barrier into the blood after oral ingestion. One hundred and fifty minutes before sacrifice, young fasted male rats received orally a solution of 1 mL of FITC Dextran (60 mg/100 g bw). Plasma FITC-dextran levels were estimated in plasma blood by fluorometric determination using GloMax Microplate Reader (Promega, Charbonnières-les-Bains, France). The sample emission spectrum from 480 nm to 600 was recorded upon excitation at 475 nm using a second-derivative spectroscopy which is a useful technique for eliminating background noise and light scattering. The FITC dextran concentration corresponded to the height of the peak detected at 514 nm. To perform this measurement, a calibration curve of FITC dextran ranging from 0.00 to 1.17 µg/µL was realized. Results were expressed as ng/mL plasma circulating FITC.

#### Gene expression of proteins involved in gut barrier permeability by reverse transcription polymerase chain reaction (RT-PCR)

Total RNA was isolated from digestive tract samples using the FastGene Basic RNA kit (NIPPON Genetics, Dutscher, Brumath, France). Its concentration was measured spectrophotometrically using a Nanodrop 1000 Spectrophotometer (Life Technologies, Thermo Fisher Scientific, Courtaboeuf, France). Complementary DNA (cDNA) was generated by reverse transcription of 1 µg mRNA with the High-Capacity cDNA Reverse Transcription Kit with RNase Inhibitor (Life Technologies, Thermo Fisher Scientific, Courtaboeuf, France) as previously described^11,27^. Expression of genes coding for tight junctions (TJ) proteins: occludin (*Ocln*), claudin 4 (*Cldn4*) and TJ Protein 1 (*Tjp1)* coding for Zonula occludens protein-1 (*ZO-1*), was measured by quantitative real-time polymerase chain reactions (RT-PCRs) (Supplementary Table S2).

Primers were purchased from Life Technologies (Life Technologies, Thermo Fisher Scientific, Courtaboeuf, France) and SYBR Green Master Mix (Life Technologies, Thermo Fisher Scientific, Courtaboeuf, France) was used. PCRs were run on a 7900HT Fast Real-Time PCR System (Applied Biosystems, 850 Lincoln Centre Drive, Foster City, California 94404, USA). Reactions were performed in duplicate.

Four genes were tested in the same conditions to keep the most robust housekeeping genes for each intestinal section: glyceraldehyde 3-phosphate dehydrogenase (*Gapdh*), hypoxanthine guanine phosphoribosyl transferase 1 (*Hprt1*), Ubiquitin C (*Ubc*) and beta actin (*Actb)*. Expression stability was determined by RefFinder^46^ using the BestKeeper program^47^, Normfinder program^48^ and Genorm program^49^ and the comparative delta-Ct method^50^. mRNA amounts were estimated using the 2-ΔΔCT method (ΔΔCt = ΔCt_exposed_–mean ΔCt_control_) compared to *Gapdh*, considered as the best housekeeping gene for jejunal and ileal segments, and *Hprt1* for proximal colon, after comparing the results of the 4 programs used.

### Evaluation of intestinal innate immune modifications in male offspring from mothers submitted to CPF and/or HFD

#### Measure of systemic markers of inflammation

##### Plasma lipopolysaccharides translocation from intestinal lumen

Plasma LPS is a useful marker for identification of increased intestinal permeability and thus of intestinal injury. Plasma assay was performed with the Rat Lipopolysaccharide ELISA kit according to the manufacturer instructions (#CSB E14247r, CliniSciences, Nanterre, France).

##### Secretion of pro-inflammatory circulating cytokines

Measurement of plasma concentration of 3 major circulating pro-inflammatory cytokines: Interleukin 1 beta (IL-1β), Interleukin 6 (IL-6) and tumor necrosis factor alpha (TNF-α) were performed using DuoSet ELISA Kit according to the manufacturer instructions (respectively DY501, DY506, DY510—ELISA Kits; Bio-techne, Lille, France).

#### Gene expression of proteins involved in innate immune protection

Briefly and as already stated above, cDNA generated from extracted mRNA was amplified according to the conditions described in paragraph 2.3.2. Expression of genes coding for innate immunity proteins: (1) Defensin β1 (*Defb1*), Defensin β4 (*Defb4*), Toll Like Receptor 2 (*Tlr2*) and Toll Like Receptor 4 (*Tlr4*) and (2) mucins proteins: Mucin 1 (*Muc1*), Mucin 2 (*Muc2*) and Mucin 3 (*Muc3*) were measured by quantitative real-time polymerase chain reactions (RT-PCRs) (Supplementary Table S2) according to the conditions already mentioned. Reactions were also performed in duplicate.

#### Histological evaluation of intestinal goblet cells number and mucus secretion alterations

Jejunal, ileal and colonic tissue sections were fixed immediately after sacrifice into a Carnoy solution (60% absolute alcohol, 30% chloroform and 10% acetic acid) for 48 h before being embedded into xylene and then paraffin. Samples were then sliced into 5 μm sections and transferred to glass slides in order to proceed to staining. The Periodic Acid Schiff (PAS) staining was used to highlight the presence of some carbohydrates in the digestive system as described below (mucins, basement membranes, glycogen as well as filaments and mycelial spores) thanks to a specific kit (395B-1KT, Sigma-Aldrich, Merck KGaA, Darmstadt, Germany). Photos were taken using a camera attached to the microscope (Olympus DP-10 Digital Camera; Olympus America, New York) and are presented in Supplementary Table S3. Staining quantifications were performed on areas from two fields/slide (100 × magnification) with the help of the ImageJ software^51^. Mucus stain positive areas (PAS positive area) were measured in a blinded manner and mucus secretion area ratio was determined according to a previous study^52^. Results were expressed as the ratio of mucus stain positive area/villus area. The density of goblet cells per villus, expressed as number of goblet cell/mm villus was also measured^29^. Five measures per animal were made.

### Systemic growth and intestinal development from mothers submitted to CPF and/or HFD

#### Systemic growth alteration of male offspring

Male pups were weight throughout the observation study from PND3 to PND54 as above stated. Three important time points were particularly observed: the neonatal time point (PND3), weaning (PND21) and the end of the experiment (PND54) in order to evaluate any alteration of the offspring weight gain depending on their mother exposure to CPF and/or HDF versus non-exposed offspring.

#### Modification of offspring intestinal and colonic layers thicknesses

On PAS stained 5 µm sections, thickness of the different intestinal layers was measured in both the intestinal and colic segments (jejunum, ileum and proximal colon). We determined the villi area and the thicknesses of the mucosa, submucosa, circular and longitudinal muscle layers. Photos were taken using a camera attached to the microscope (Olympus DP-10 Digital Camera, Olympus America, New York). Measures from two fields/slide (100 × magnification) and from five measures per animal were also analysed in a blinded manner with the help of the ImageJ software^51^. Results were expressed as µm^2^ for villi area, and µm for thicknesses of the mucosa, submucosa, circular and longitudinal muscle layers.

### Statistical analysis

Data were expressed as mean ± Standard Error to the Mean (SEM) and were analyzed using GraphPad Prism software (GraphPad Prism version 8.4.3 for Windows, GraphPad Software, San Diego, California USA, www.graphpad.com) Non-parametric Kruskal–Wallis tests were performed and when the difference was significant, a Dunn’s post-hoc test was further realized with n = 16 for offspring ctrl-SC group; n = 20 for offspring CPF-SC group; n = 10 for offspring ctrl-HF group: maternal exposure to HFD without CPF and n = 5 for offspring CPF-HF group. A value of p < 0.05 was considered significant.

## Supplementary Information

Supplementary Information.

## Data Availability

The datasets generated during and/or analyzed during the current study are available from the corresponding author on reasonable request.

## Abbreviations

AChE: Acetylcholinesterase
*Actb*: Beta actin
BW: Body weight
cDNA: Complementary DNA
*Cldn4*: Claudin 4
CPF: Chlorpyrifos
d: Day
*Defb1*: Defensin β1
*Defb4*: Defensin β4
FITC: Fluorescein isothiocyanate
*Gapdh*: Glyceraldehyde 3-Phosphate dehydrogenase
HFD: High fat diet
*Hprt1*: Hypoxanthine guanine phosphoribosyl transferase 1
IL-1β: Interleukin 1 beta
IL-6: Interleukin 6
LPS: Lipopolysaccharide
*Muc1*: Mucin 1
*Muc2*: Mucin 2
*Muc3*: Mucin 3
NOAEL: No observable adverse effect level
*Ocln*: Occludin
OPs: Organophosphates
PAS: Periodic acid schiff
PND: Postnatal day
RT-PCR: Reverse transcription polymerase chain reaction
SCD: Standard chow diet
SEM: Standard error to the mean
TJ: Tight junctions
*Tjp1*: Tight junction protein 1
*Tlr2*: Toll like receptor 2
*Tlr4*: Toll like receptor 4
TNF-α: Tumor necrosis factor alpha
*Ubc*: Ubiquitin C
Zo-1: Zonula occludens protein-1
