# Supplementary material for: Programming of intestinal homeostasis in male rat offspring after maternal exposure to chlorpyrifos and/or to a high fat diet
Source: Sci Rep. 2021 Jun 1;11:11420. doi: 10.1038/s41598-021-90981-2 (PMC8169651; doi:10.1038/s41598-021-90981-2)
Supplement: Supplementary file 1 — Supplementary Information. [file 41598_2021_90981_MOESM1_ESM.docx]

**Programming of intestinal homeostasis in male rat offspring after maternal exposure to chlorpyrifos and/or to a high fat diet**

Marion Guibourdenche, MSc ^1, 2^; Hiba El Khayat El Sabbouri, PhD ^1^; Narimane Djekkoun, MSc ^1^; Hafida Khorsi-Cauet, PhD ^1^; Véronique Bach, PhD ^1^; Pauline M Anton, PhD ^2^; Jérôme Gay-Quéheillard, PhD ^1,^ *.

^1^ PériTox, Périnatalité & Risques Toxiques, UMR-I 01 INERIS, Université Picardie Jules Verne, Amiens, France

^2^ Institut Polytechnique UniLaSalle, Université d’Artois, URL 7519, 19 rue Pierre Waguet, BP 30313, 60026 Beauvais, France

* Corresponding author : Dr. Jérôme Gay-Quéheillard, [Jerome.gay@u-picardie.fr](mailto:Jerome.gay@u-picardie.fr), PériTox, Périnatalité & Risques Toxiques, UMR-I 01 INERIS , Université Picardie Jules Verne, Présidence, CURS, Chemin du Thil, 80025 Amiens

**Supplementary Table S1- Composition of the different rodent chows used during the experiment**

| **Crude nutrients %** | **HFD ^a^** | **Standard chow diet ^b^** | **Breeding standard diet ^c^** |
| --- | --- | --- | --- |
| **Type** | D12492 | 3436 | 3336 |
| **Supplier** | Ssniff Spezialdiäten GmbH. Soest. Germany | SERLAB Montataire  France | SERLAB Montataire  France |
| **Aim** | High calorie diet | Maintenance | Pregnancy/ lactation |
| **Gross Energy (GE) MJ/kg** | 25 | 16.1 | 17 |
| **Metabolizable energy (ME) MJ/kg** | 21.6 | 13.1 | 14.3 |
| **Metabolizable energy density (Kcal/g)** | 5.16 | 3.13 | 3.41 |
| **Major nutrients (%)** | | | |
| **Crude protein** | 24.4 | 18.5 | 23.5 |
| **Crude fat** | 34.6 | 4.5 | 5.5 |
| **Crude fiber** | 6.0 | 4.5 | 3.0 |
| **Crude ash** | 5.3 | 6.5 | 5.7 |
| **Starch** | 0.1 | 35 | 36 |
| **Sugar** | 9.4 | Unkown | Unkown |
| **N free extracts (NFE)** | 26.3 | 54 | 51.3 |
| **Minerals (%)** | | | |
| **Calcium** | 0.92 | 1.05 | 1.00 |
| **Phosphorus** | 0.64 | 0.80 | 0.74 |
| **Magnesium** | 0.23 | 0.20 | 0.20 |
| **Sodium** | 0.20 | 0.20 | 0.20 |
| **Potassium** | 0.97 | 0.78 | 0.80 |
| **Chlorine** | Unkown | 0.36 | 0.33 |
| **Amino acids (%)** | | | |
| **Arginine** | 0.95 | 1.10 | 1.50 |
| **Lysine** | 2.02 | 1.00 | 1.40 |
| **Methionine** | 0.86 | 0.39 | 0.60 |
| **Methionine + cysteine** | 1.31 | 0.76 | 0.60 |
| **Tryptophan** | 0.33 | 0.20 | 0.27 |
| **Threonine** | 1.07 | 0.65 | 0.87 |
| **Vitamins (/kg)** | | | |
| **Vitamin A** | 15,000 IU | 14,000 IU | 14,000 IU |
| **Vitamin D3** | 1,500 IU | 1,000 IU | 1,000 IU |
| **Vitamin E** | 150 mg | 110 mg | 110 |
| **Vitamin K** | 20 mg | 2 mg | 2 mg |
| **Thiamine (B1)** | 25 mg | 30 mg | 32 mg |
| **Riboflavin (B2)** | 16 mg | 20 mg | 21 mg |
| **Pyridoxine (B6)** | 16 mg | 14 mg | 14 mg |
| **Cobalamin (B12)** | 30 µg | 50 µg | 50 µg |
| **Nicotinic acid** | 47 mg | 70 mg | 70 mg |
| **Panthothenic acid** | 55 mg | 33 mg | 33 mg |
| **Folic acid** | 16 mg | 2 mg | 2.4 mg |
| **Biotin** | 300 µg | 220 µg | 220 µg |
| **Choline** | 920 mg | 2000 mg | 2000 mg |
| **Vitamin C** | Unknown | 40 mg | 40 mg |
| **Trace elements (mg/kg)** | | | |
| **Iron** | 168 | 250 | 200 |
| **Zinc** | 65 | 60 | 68 |
| **Copper** | 13 | 14 | 15 |
| **Iodine** | 1.2 | 1 | 1 |
| **Manganese** | 95 | 60 | 40 |
| **Selenium** | 0.2 | 0.3 | 0.27 |

a. Contains (in g/kg to total 1000) : Pork lard (315), Casein (277), Maltodextrin (156), Sucrose (85), Cellulose powder (60), Mineral premix (60), Soybean oil (31), Vitamin premix (10), L-Cystine (3.5), Choline chloride (2.5), Dye (blue)(0.3), Butylated hydroxytoluene (0.1), Corn starch (0)

Fatty acid composition of HFD : Lauric acid (0.07%), Myristic acid (0.44%), Palmitic acid (7.93%), Stearic acid (4.37%), Arachidic acid (0.11%), Linolenic acid (0.49%), Linolelaidic acid (4.64%), Palmitoleic acid (0.94%), Oleic acid (13.97%)

b. Contains : Wheat middlings, wheat, barley, soybean meal (NGMO), corn (NGMO),poultry meal, wheat starch, whey powder, soybean oil (2.13%), sunflower cake, brewer’s dried yeast, minerals, vitamins, amino acids

c. Contains : Flacked oats, soybean meal (NGMO), poultry meal, wheat middlings, wheat starch,barley, herring meal, grass meal, whey powder, corn (NGMO), soybean oil, minerals, vitamins, amino acids

**Supplementary Table S2 - Description of genes studied**

|  |  | **Name** | **FW** | **RW** | **Tm** | **Ref** |
| --- | --- | --- | --- | --- | --- | --- |
| **HOUSEKEEPING GENES** | ***Gapdh*** | *Glyceraldehyde 3 phosphate dehydrogenase* | AGACAGCCGCATCTTCTTGT | CTTGCCGTGGGTAGAGTCAT | 60 | **NM_017008.4**  [34] |
|  | ***Actb*** | *Beta actin* | ACGTCGACATCCGCAAAGACCTC | TGATCTCCTTCTGCATCCGGTCA | 60 | [35] |
|  | ***Ubc*** | *Ubiquitin C* | TCGTACCTTTCTCACCACAGTATCTAG | GAAAACTAAGACACCTCCCCATCA | 60 | **NM_017314.1**  [36] |
|  | ***Hprt1*** | *hypoxanthine guanine phosphoribosyl transferase 1* | CTCATGGACTGATTATGGACAGGAC | GCAGGTCAGCAAAGAACTTATAGCC | 60 | **NM_012583**  [37] |
| **TIGHT JUNCTIONS** | ***Ocln*** | *Occludin* | CACGTTCGACCAATGC | CCCGTTCCATAGGCTC | 58 | **XM_032898729.1**  [38] |
|  | ***Cldn4*** | *Claudin 4* | GTGCCTGGAGTCTTGGTGTC | GAGGGTAGGTGGGTGGGTAA | 58 | **NM_001012022.1**  [39] |
|  | ***Tjp1*** | *Tight junction protein 1* | GTATCCGATTGTTGTGTTCC | TCACTTGTAGCACCATCCGC | 58 | **XM_218747**  [40] |
| **IMMUNITY** | ***Defb1*** | *Defensin β1* | GGACGCAGAACAGATCAATACCGA | TCTTCAAACCACTGTCAACTCCTG | 60 | **NM_031810.1**  [41] |
|  | ***Defb4*** | *Defensin β4* | TTAATTTGGTTTGTTTTGTGCAT | CATGCCTGACCAAAGGAGGCGTA | 58 | **NM_022544.2**  [41] |
|  | ***Tlr2*** | *Toll Like Receptor 2* | GCACTTGAGCGAGTCTGCTTTC | GAACAAATAGAATCGGGGGATGTG | 60 | **NM_198769.2** [41] |
|  | ***Tlr4*** | *Toll Like Receptor 4* | GGCTGTGGAGACAAAAATGACCTC | AGGCTTGGGCTTGAATGGAGTC | 60 | **NM_019178.1**  [41] |
| **MUCINS** | ***Muc1*** | *Mucin 1* | GAGTGAATATCCTACCTACCAC | TTCACCAGGCTAACGTGGTGAC | 58 | **XM_032897995.1**  [42] |
|  | ***Muc2*** | *Mucin 2* | GCCAGATCCCGAAACCA | TATAGGAGTTCCGGCAGTCA | 60 | **NM_023566.4**  [42] |
|  | ***Muc3*** | *Mucin 3* | AACTTCCAGCCCTCCCTAAG | GCTTCCAGCATCGTCTCTCT | 60 | **U76551.1** [42] |

**Supplementary Table S3- PAS staining on digestive segments in male offspring (8 weeks old) from CPF and/or HFD exposed mothers.**

|  | **Jejunum** | **Ileon** | **Colon** |
| --- | --- | --- | --- |
| **ctrl-SC** | 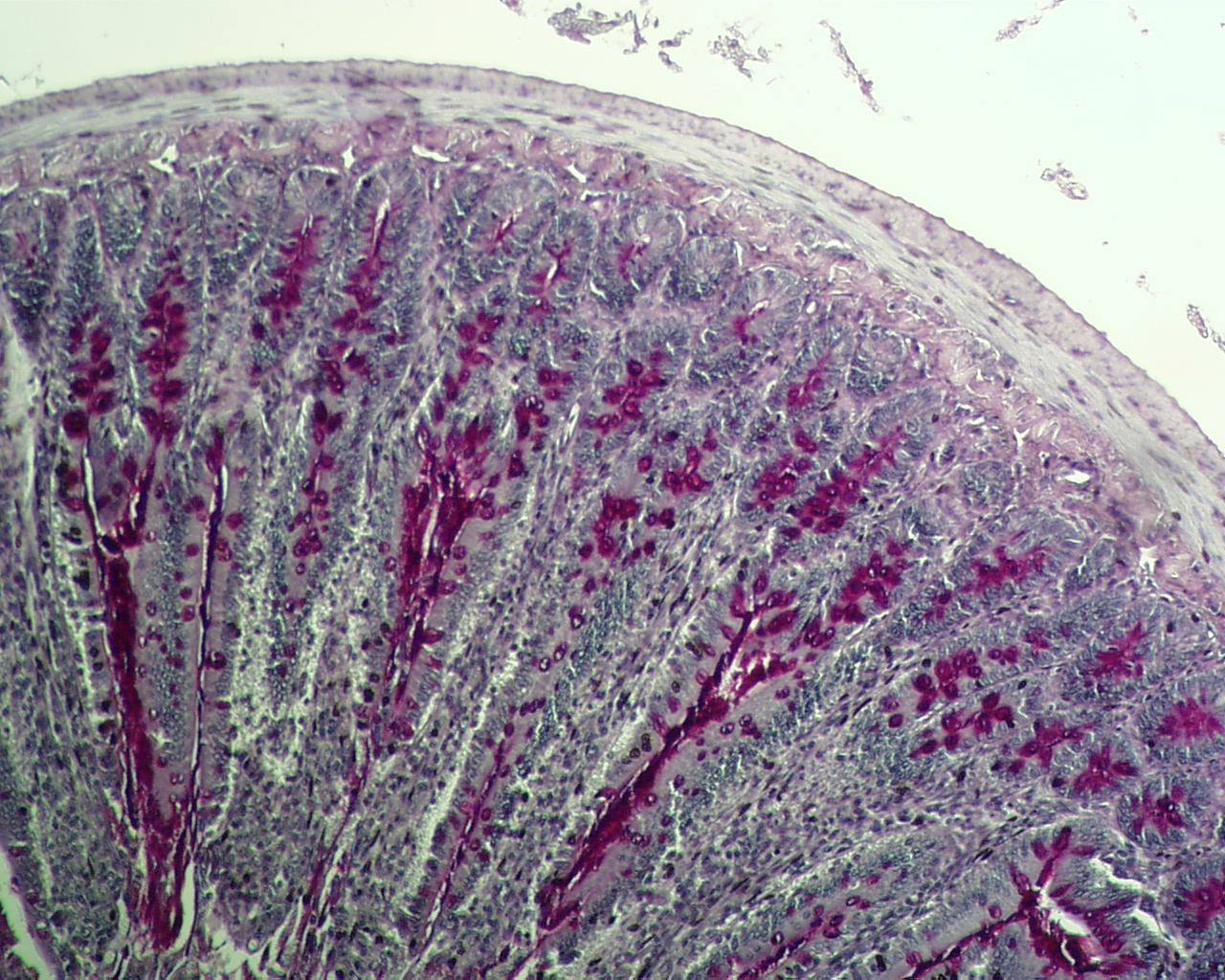 | 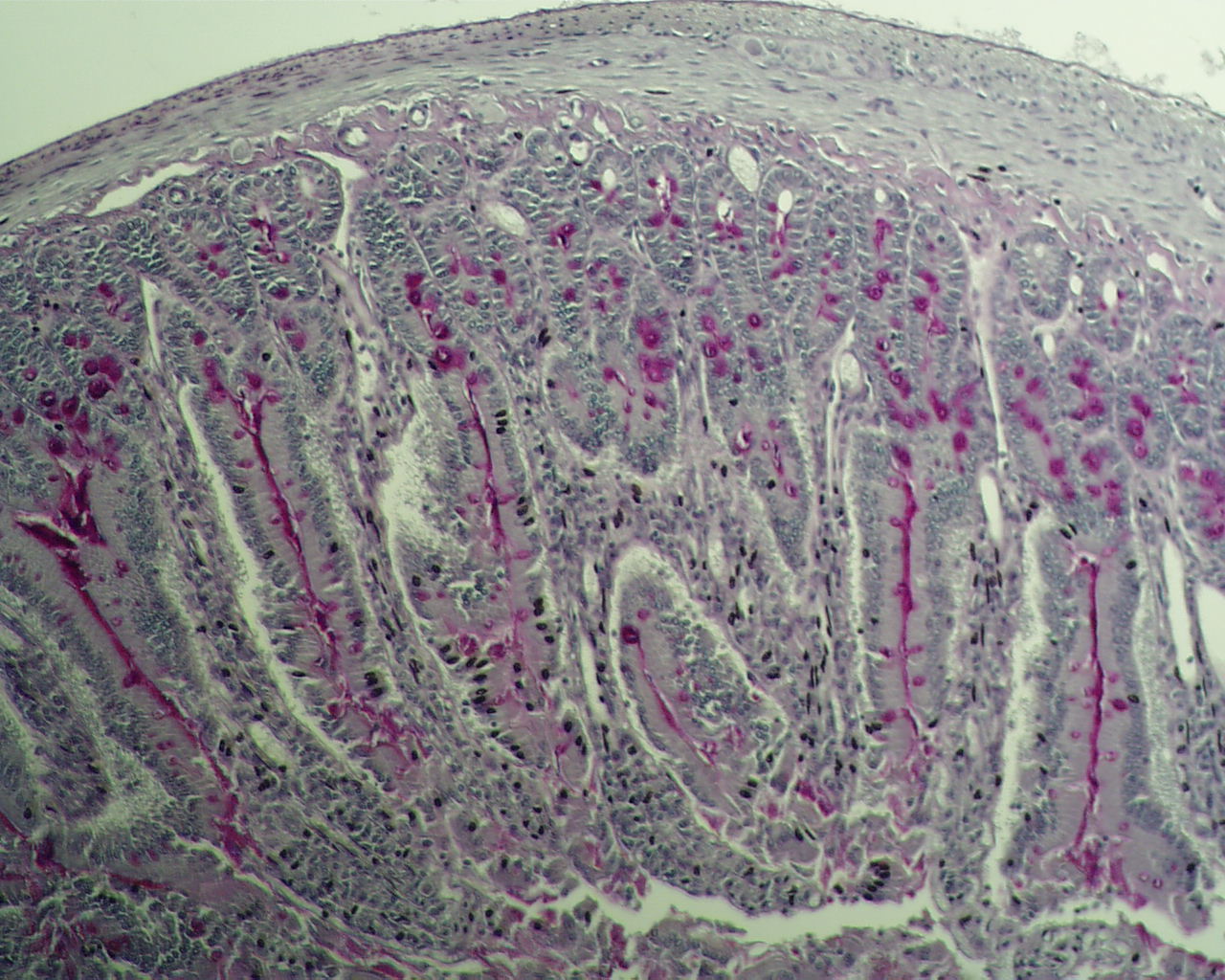 | 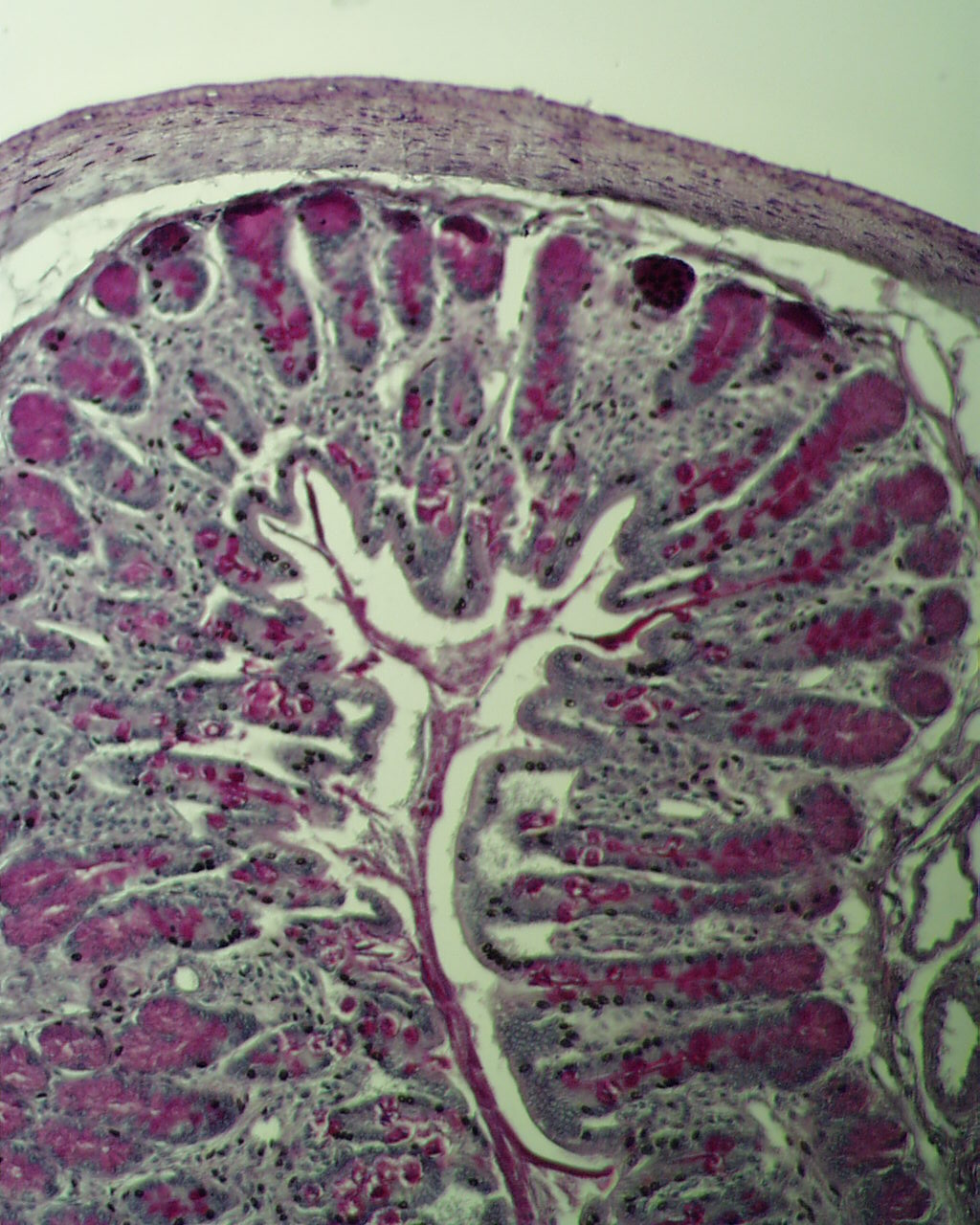 |
| **CPF-SC** | 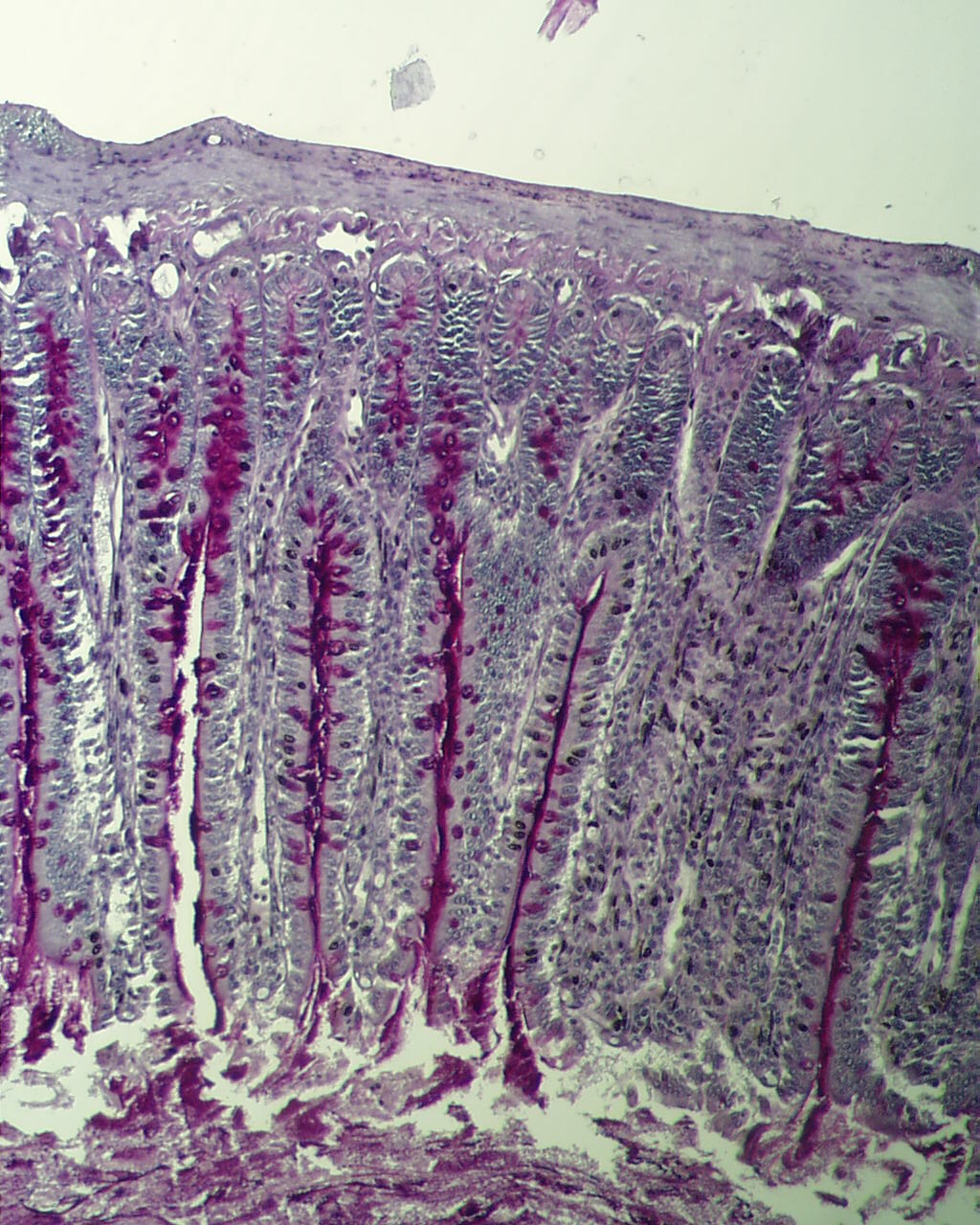 | 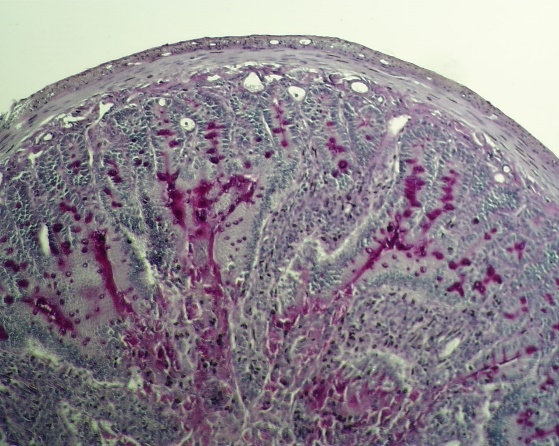 | 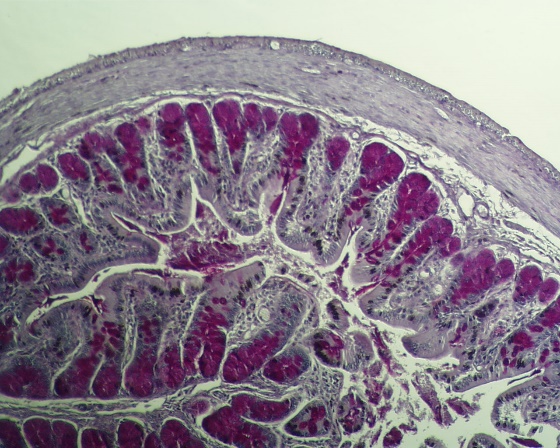 |
| **ctrl-HF** | 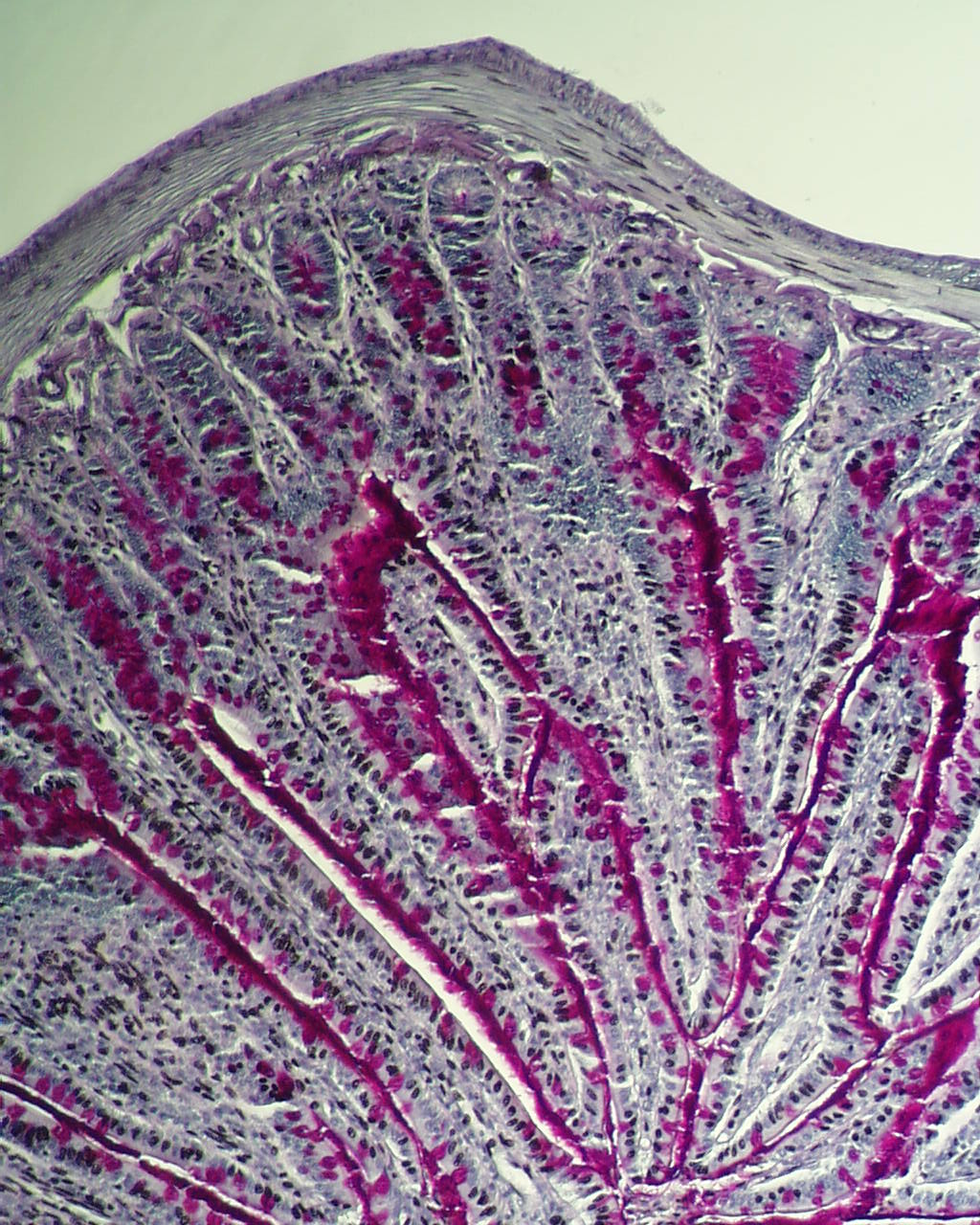 | 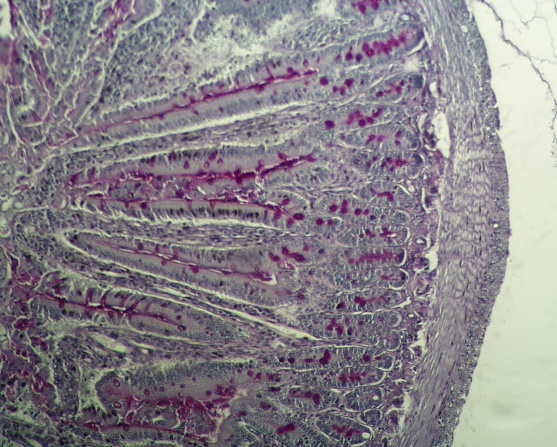 | 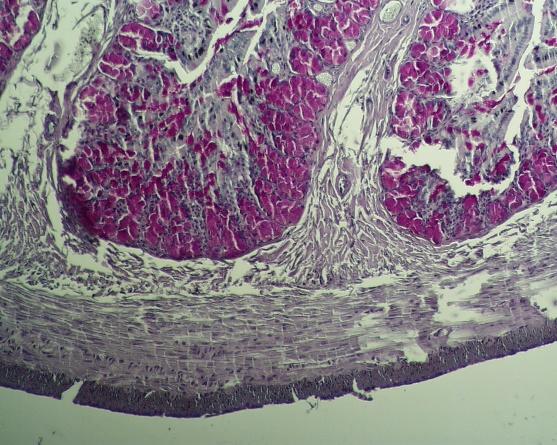 |
| **CPF-HF** | 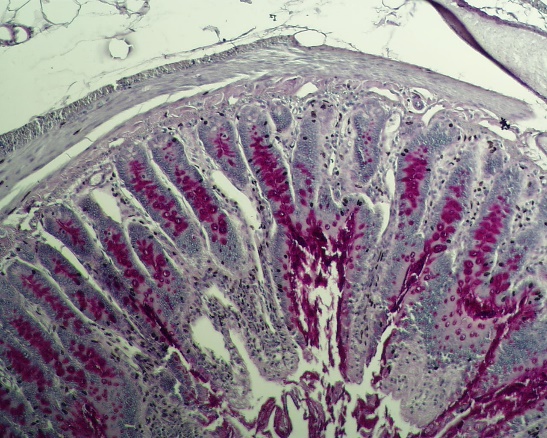 | 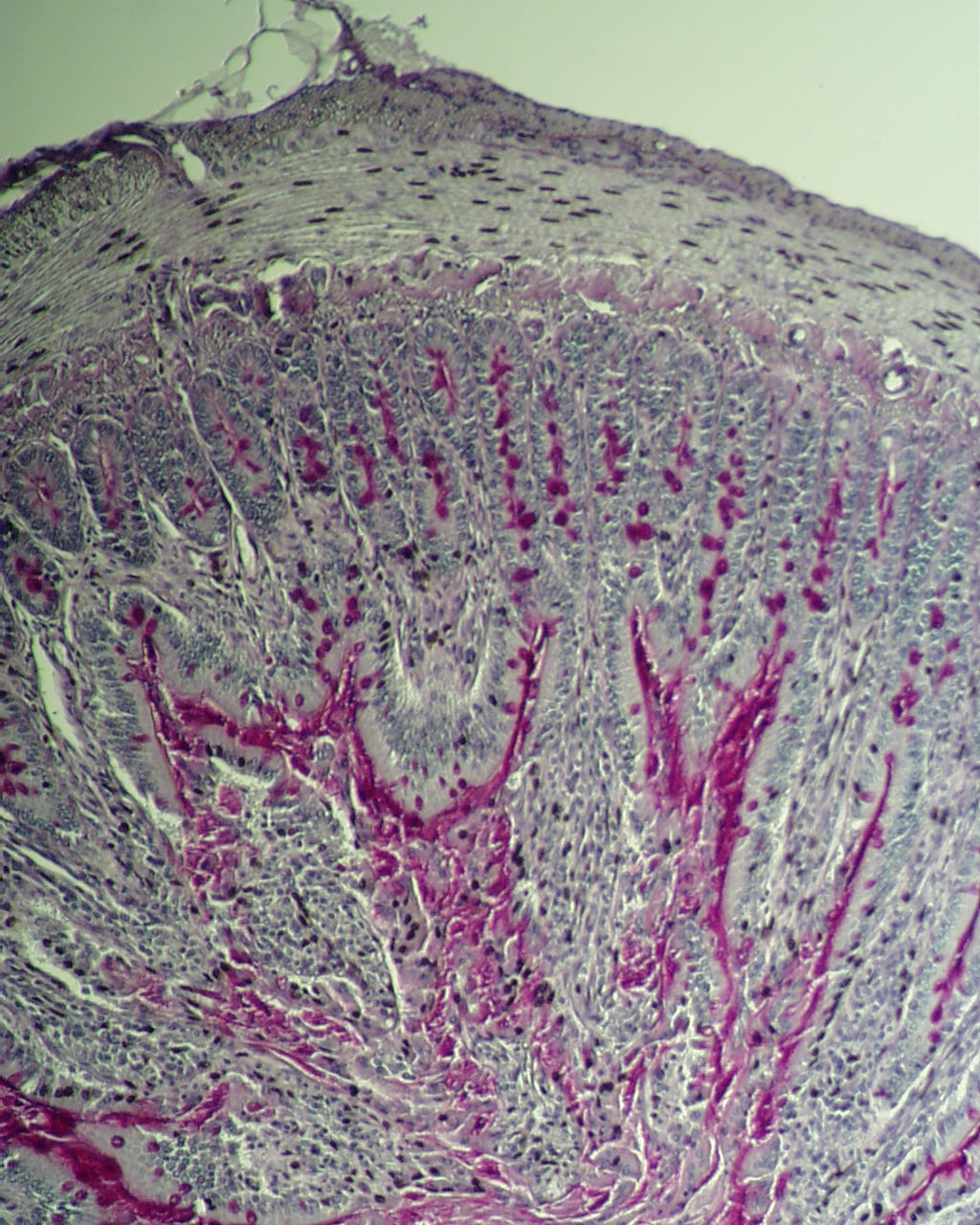 | 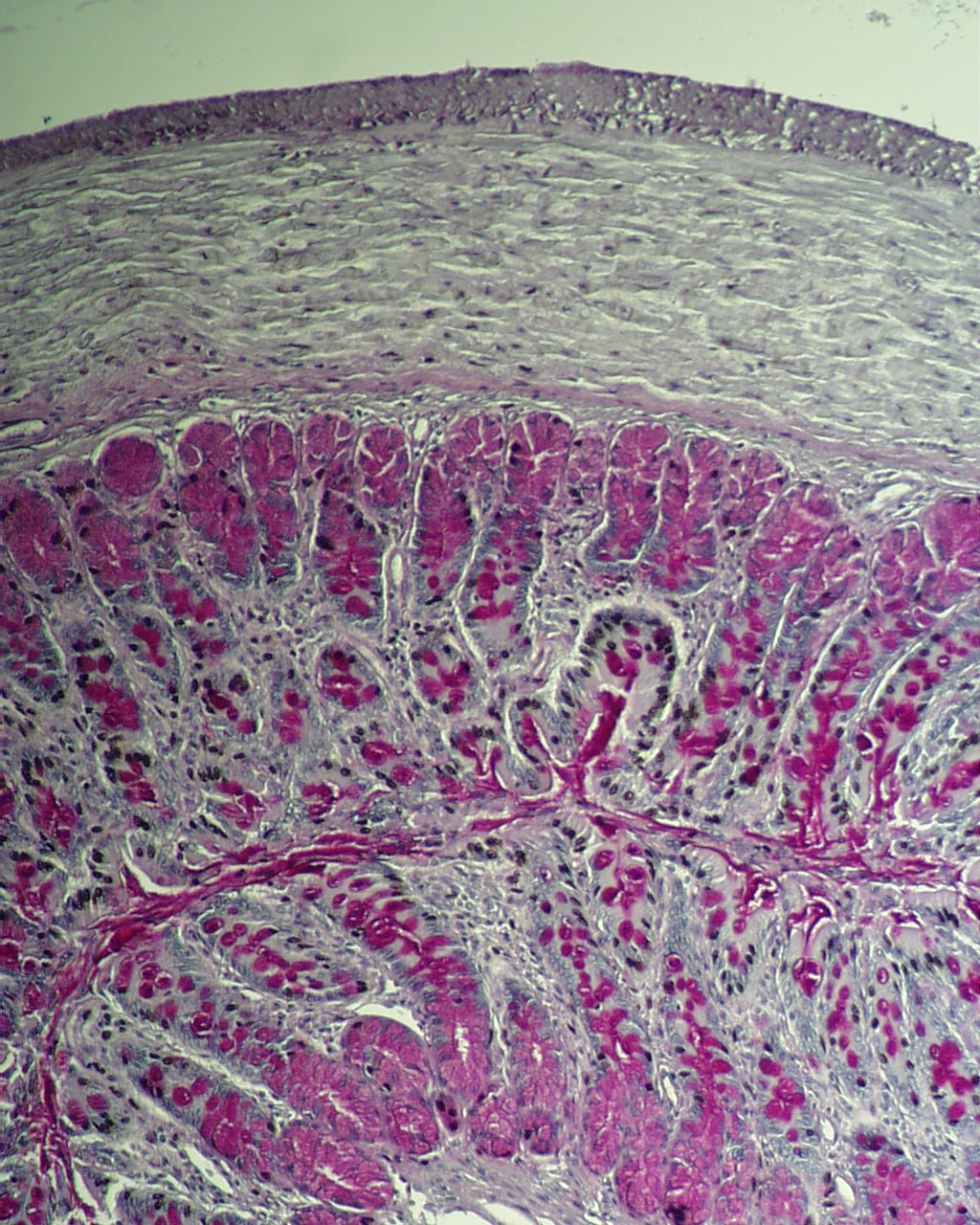 |

PAS staining visualized at magnification 100x. CPF; Chlorpyrifos; ctrl, control; HF, High Fat; PAS; Periodic Acid Schiff; SC, Standard Chow.

**Supplementary Table S4 – Daily food consumption (grams) of male offspring from CPF and/or HFD exposed mothers**

|  | ctrl-SC | CPF-SC | ctrl-HF | CPF-HF |
| --- | --- | --- | --- | --- |
| PND24-PND31 | 11,72 ± 0,9 | 12,82 ± 0,5 | 12,83 ± 0,4 | 13,33 ± 0,1 |
| PND31-PND38 | 16,52 ± 1 | 16,65 ± 0,5 | 18,01 ± 0,5 | 16,15 ± 0,1 |
| PND38-PND45 | 16,37 ± 1,3 | 21,78 ± 0,5 | 21,00 ± 0,7 | 23,90 ± 0,8 |
| PND45-PND52 | 25,33 ± 0,4 | 25,82 ± 1,5 | 21,63 ± 1,2 | 23,33 ± 0,7 |

The mean weekly food intake / rat / day starting after weaning and ending at the end of the experiment. Values are expressed as mean ± SEM (n = 5-20) using the analysis of variance (ANOVA) and a subsequent post hoc Bonferroni test. ctrl, control; CPF, Chlorpyrifos; HF, High Fat; SC, Standard Chow; PND, Postnatal Day.
